# Exploring the pharmacokinetics, drug-likeness, and toxicological features of anticancer flavonoids: a Boulevard to explore their clinical translational potential

**DOI:** 10.3389/fphar.2025.1648395

**Published:** 2025-10-03

**Authors:** Ankit Kumar Dubey, Siva Sai Chandragiri, Abin V. Geevarghese, Bhupinder Kapoor, Monica Gulati, Pooja Rani, Gursharan Singh, Vivek P. Chavda, Rohit Gundamaraju, Himangini Bansal, Rupesh K. Gautam, Rajat Goyal, Michael P. Okoh, Bairong Shen, Rajeev K. Singla

**Affiliations:** ^1^ Department of Pharmacy and Institutes for Systems Genetics, Center for High Altitude Medicine, Frontiers Science Center for Disease-related Molecular Network, West China Hospital, Sichuan University, Chengdu, Sichuan, China; ^2^ iGlobal Research and Publishing Foundation, New Delhi, India; ^3^ Department of Pathology, University of Oklahoma Health Sciences Center, Oklahoma City, OK, United States; ^4^ Department of Pharmacology, Faculty of Pharmacy, Karpagam Academy of Higher Education, Coimbatore, Tamil Nadu, India; ^5^ School of Pharmaceutical Sciences, Lovely Professional University, Phagwara, Punjab, India; ^6^ Faculty of Health, Australian Research Centre in Complementary and Integrative Medicine, University of Technology Sydney, Sydney, NSW, Australia; ^7^ Department of Medical Laboratory Sciences, Lovely Professional University, Phagwara, India; ^8^ Department of Pharmaceutics and Pharmaceutical Technology, L M College of Pharmacy, Ahmedabad, Gujarat, India; ^9^ ER stress and mucosal immunology lab, School of Health Sciences, University of Tasmania, Launceston, TAS, Australia; ^10^ School of Medicine, University of California, San Francisco, San Francisco, CA, United States; ^11^ Department of Pharmaceutical Engineering, B V Raju Institute of Technology, Medak, Telangana, India; ^12^ Delhi Institute of Pharmaceutical Sciences and Research, New Delhi, India; ^13^ IES Institute of Pharmacy, IES University, Bhopal, Madhya Pradesh, India; ^14^ Amity Institute of Pharmacy, Amity University Uttar Pradesh, Noida, India; ^15^ MM College of Pharmacy, Maharishi Markandeshwar (Deemed to be) University, Mullana-Ambala, Haryana, India; ^16^ Department of Medical Biochemistry, Faculty of Basic Medical Sciences, College of Health Sciences, University of Abuja, Abuja, Nigeria

**Keywords:** flavonoids, anticancer agents, pharmacokinetics, admet, drug toxicity

## Abstract

**Background:**

Flavonoids that are widely distributed across various plant species exhibit significant anticancer activity in various preclinical and clinical studies, thus offering promising therapeutic prospects. However, a thorough understanding of their pharmacokinetic properties, drug-likeness characteristics, and safety profile is essential for the translational applicability of these molecules into clinical settings.

**Methods:**

A systematic search was carried out using various electronic databases such as PubMed Central, ScienceDirect, Clinical Registry, and Google Scholar, using different keywords like “flavonoids”, “cancer”, “pharmacokinetics”, “toxicity”, “tumor”, and their combinations. Non-English literature was excluded due to language barriers, limited accessibility, non-indexing, and the risk of misinterpreting methods or results, which could compromise the accuracy and reliability of the review.

**Results and discussion:**

This review provides an in-depth overview of various mechanistic pathways, such as oxidative stress-mediated and immunomodulatory pathways, that are considered to be responsible for the anti-cancer potential of flavonoids. In addition, the pharmacokinetic properties and toxicity profile of flavonoids have been discussed, which are the crucial factors in their clinical translation. Lastly, the review briefly explores various strategies that can be adopted to improve the effectiveness of flavonoids in the treatment of cancer.

**Conclusion:**

This investigation enhances our understanding of the translational potential of flavonoid-based therapies by highlighting these essential elements, bringing us one step closer to the development of effective and safe cancer treatments.

## Graphical Abstract

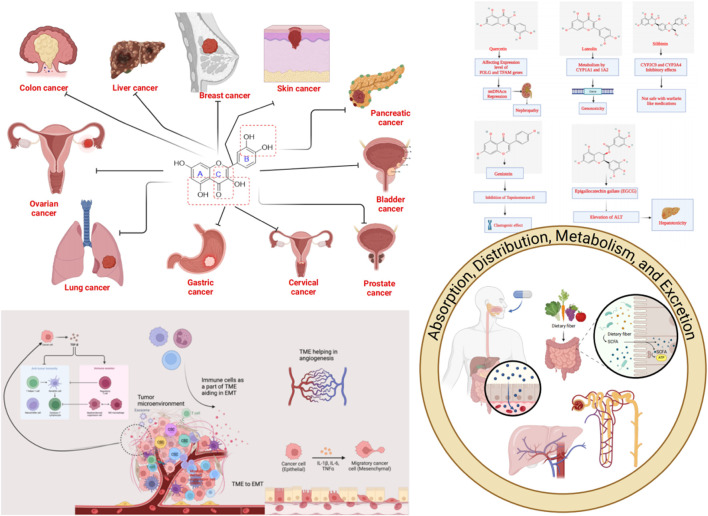

## 1 Introduction

**FIGURE 1:**
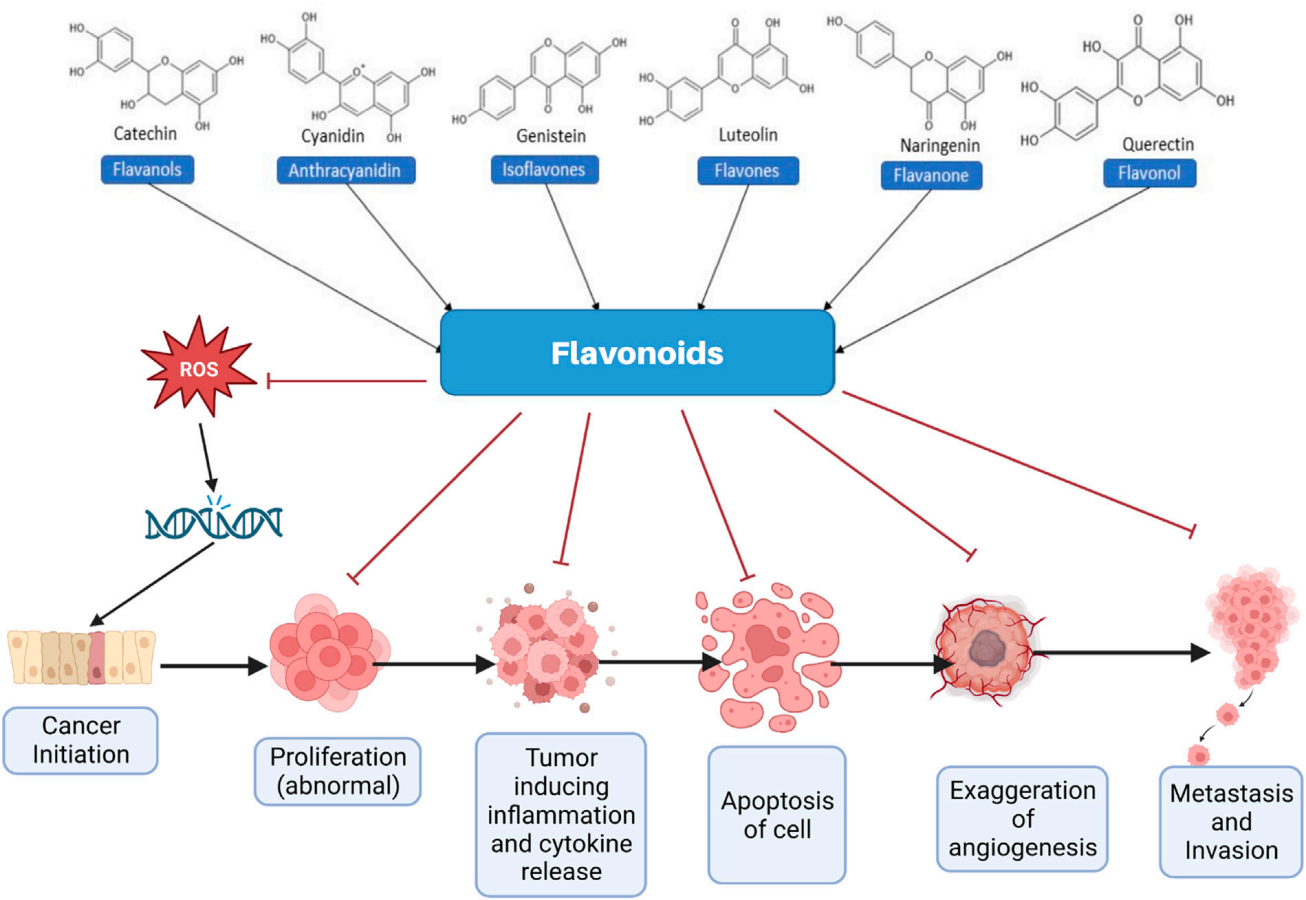
Different types of flavonoids and their role in effectively managing cancer. ROS, Reactive oxygen species.

Flavonoids are naturally occurring secondary metabolites that can be obtained from various plant parts such as fruits, vegetables, roots, stems, flowers (Zheng et al., 2025). They function in plants as light filters, visual attractants, feeding repellents, antioxidants, antimicrobials, and photoreceptors (Pietta, 2000). These properties underpin their wide applicability in cosmetics and medicine (Kurek-Gorecka et al., 2020). Flavonoids also have diverse therapeutic applications in humans due to their antioxidant, anticancer, antiviral, anti-inflammatory, and anti-allergic properties (Pietta, 2000; Chavda et al., 2022). Chemically, flavonoids are the polyphenolic compounds that can be classified as flavanols, isoflavonoids, anthocyanidins, flavones, and flavonols (Figure 1) (Panche et al., 2016).

Flavonoids are widely being recognized as potent anticancer agents that exert their effects through various mechanisms, including scavenging of reactive oxygen species (ROS), promoting apoptosis, inducing cell cycle arrest, anti-angiogenesis, and inhibiting the proliferation and invasion of cancer cells (Pietta, 2000; Rana and Mumtaz, 2025). The hydroxyl groups present in flavonoids are primarily responsible for their therapeutic activities, particularly their antioxidant and ROS-scavenging properties (Vo et al., 2019). Consumption of a flavonoid-rich diet is reported to decrease the cancer risk in various studies (Rodriguez-Garcia et al., 2019; Chavda et al., 2021; Khan et al., 2021; Parmenter et al., 2025). Moreover, various flavonoids, including quercetin, kaempferol, and morin, effectively modulate cancer cell chemoresistance by inhibiting efflux transporters such as P-glycoprotein (P-gp) and multidrug resistance-associated proteins (MRPs), thereby enhancing intracellular drug accumulation and restoring drug sensitivity (Liskova et al., 2021). Additionally, flavonoids modulate resistance-associated signaling pathways, including nuclear factor-κB (NF-κB), phosphoinositide 3-kinase/protein kinase B (PI3K/Akt), mitogen-activated protein kinase (MAPK), and signal transducer and activator of transcription 3 (STAT3), thereby influencing cell survival, apoptosis, and DNA repair mechanisms (Bukowski et al., 2020; Tian et al., 2023; Mir et al., 2024). Figure 1 briefly captures various inhibitory anticancer mechanisms elicited by various flavonoids, in general.

Despite immense therapeutic potential, the unfavorable pharmacokinetic properties of flavonoids limit their use in clinical settings (Hussain et al., 2025). Most flavonoids exhibit poor water solubility, low oral absorption, and undergo extensive first-pass metabolism, resulting in limited systemic bioavailability (Halevas et al., 2022). These pharmacokinetic challenges necessitate the development of innovative delivery systems such as nanoparticles, liposomes, and solid lipid carriers to enhance their stability, absorption, and targeted delivery of flavonoids (Billowria et al., 2024; Hu et al., 2025). Nanoparticle system loaded with flavonoids is reported to increase the drug retention time, thereby enhancing their circulation time and stability at a lower drug dose (Sharma et al., 2022; Abdi Syahputra et al., 2024; Rana and Mumtaz, 2025). Furthermore, structural modification using a prodrug strategy has been explored to introduce favorable physicochemical properties that enhances chemical stability and target specificity of flavonoids (Zhao et al., 2019; Dehelean et al., 2022). Recently, an apigenin prodrug has been synthesized, which exhibited increased stability and cytotoxic potential as compared to the parent compound apigenin (Diamantis et al., 2023). In another study, conjugation of quercetin with amino acids is reported to increase the solubility, stability, cellular permeability, as well as anticancer activity (Dobrydnev et al., 2020). Overall, the expanding field of flavonoid pharmacology now bridges traditional phytomedicine with modern drug development tools, paving the way for clinically translatable anticancer therapeutics (Abdi Syahputra et al., 2024). This article emphasizes the exploration of the aforementioned parameters to ensure the therapeutic efficacy and safety of flavonoids in cancer treatment and management. Flavonoids are highlighted as potent natural anticancer agents with diverse mechanisms but limited clinical applications due to poor pharmacokinetics. Emerging drug delivery systems and prodrug approach aim to bridge the gap.

Rather than a catalog, this review is organized around translation bottlenecks (exposure, selectivity, and safety) and how modern solutions shrink them. Nanoparticle and nanogel carriers consistently raise bioavailability and tumor deposition while reducing off-target exposure; prodrugs and methyl/glycosyl edits rebalance permeability-metabolism tradeoffs; and drug-drug interactions (DDI)-aware scheduling mitigates CYP3A4-linked risks. We map each mechanistic promise (for example, tumor microenvironment (TME) reprogramming, efflux modulation) to an ADME (absorption, distribution, metabolism, and excretion)-compatible formulation and a clinical next step (PK/PD (pharmacokinetics/pharmacodynamics) markers, combo logic), emphasizing 2024–2025 advances (Dewanjee et al., 2023; Abdi Syahputra et al., 2024; Kondža et al., 2024; Hu et al., 2025; Liga and Paul, 2025).

## 2 Cancer and drug resistance

Cancer remains one of the leading causes of chronic disease-related deaths worldwide, accounting for nearly 10 million deaths in 2020, according to the World Health Organization (WHO). Cancer is characterized by the uncontrolled growth of cells, and can be cured if diagnosed at an early stage (Mathur et al., 2015). Currently, cancer is treated using chemotherapy, surgery, radiation therapy, immunotherapy, endocrine therapy, and targeted therapy. Among these, immunotherapy remains a promising but challenging approach. Side effects associated with cancer chemotherapy, such as immunosuppression, drug resistance, and systemic toxicities, limit the scope of chemotherapeutic agents (Aslam et al., 2014). Surgery and radiation therapy are used to treat a specific tumor or an area of the body (Baskar et al., 2012). Surgical resection causes physical pain to the patients as well as impairs the normal functioning of surrounding tissues (Jain et al., 2014; Chen et al., 2022).

**FIGURE 2:**
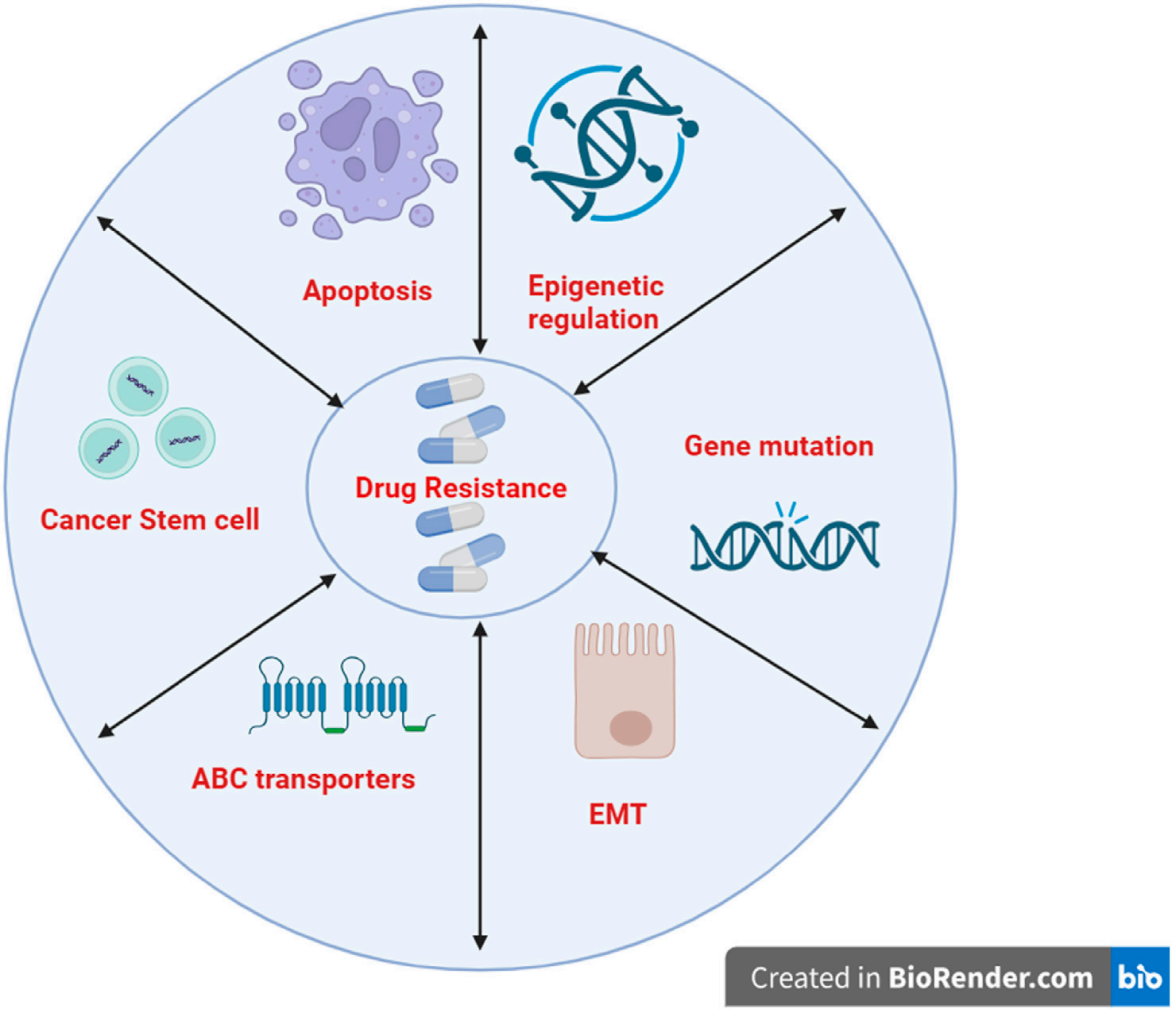
Drug resistance and cancer. Molecular mechanisms like apoptosis, epigenetic regulation, gene mutation, EMT, and ABC transporters are involved. (Created with Biorender.com). EMT, Epithelial-mesenchymal transition; ABC, ATP-binding cassette.

In clinical practice, surgery and chemotherapy are the most commonly used treatments for cancer; however, cancer cells often develop resistance to anticancer drugs over time. Drug resistance is responsible for 90% of chemotherapy failures (Davodabadi et al., 2023). Cancer cells develop resistance to cytotoxic drugs through multiple complex mechanisms, which can be intrinsic or acquired. Intrinsic resistance refers to the inherent ability of cancer cells to withstand therapeutic interventions even before treatment begins. This resistance is heterogeneous and may arise due to inherited genetic mutations that render tumor cells less responsive to chemotherapeutic agents, pre-existence of unresponsive cancer stem cells or activation of intrinsic pathways that can remove or decrease the intracellular concentration of drugs. On the other hand, acquired resistance emerges during treatment and is typically attributed to the development of drug-resistant cancer cell populations that often contain secondary genetic alterations, ultimately leading to therapy failure. Multiple mechanisms have been implicated in acquired resistance, including modulation of TME, alteration in DNA repair mechanisms, dysregulation of transmembrane transporters that reduced intracellular drug concentrations, epigenetic and epistatic modifications, inactivation of drug due to metabolism, and mutations in drug targets (Michael and Doherty, 2005; Eslami et al., 2024; Khan et al., 2024). This critical issue can ultimately result in disease recurrence, treatment failure, and increased mortality. Figure 2 illustrates the diverse factors contributing to drug resistance in cancer. Cancer drug resistance arises from both intrinsic and acquired mechanisms, complicating treatment and contributing to therapy failure. This underscores the need for alternative or adjunctive approaches.

## 3 Tumor microenvironment- an update

**FIGURE 3:**
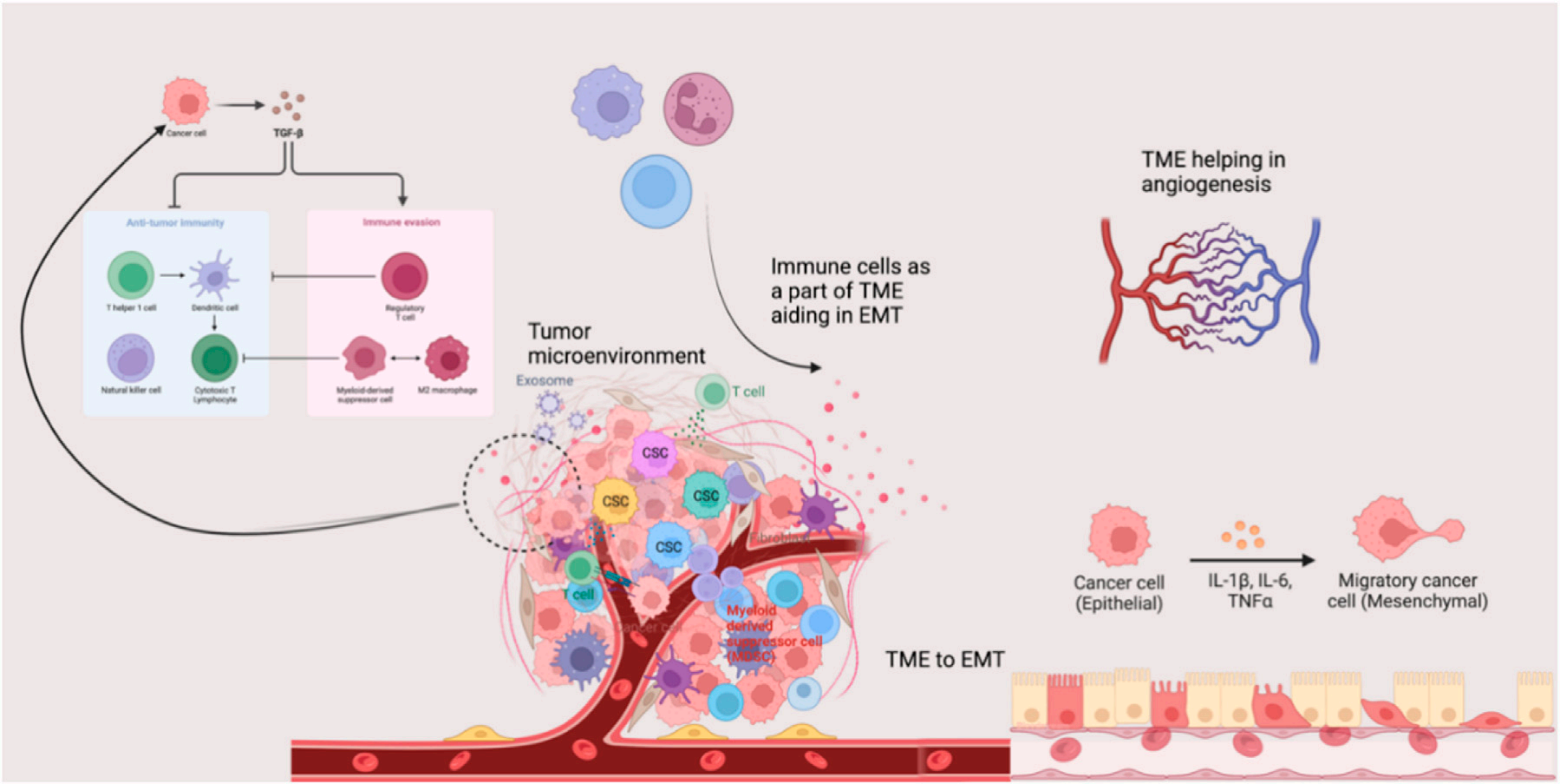
Tumor microenvironment and disease progression. The tumor microenvironment possesses several components that can orchestrate events such as angiogenesis, EMT, and aggressive cancer invasion. TME, Tissue microenvironment; EMT, Epithelial-mesenchymal transition; TGF-β, Transforming growth factor-beta; IL, Interleukin.

The TME comprises a complex and dynamic network of non-malignant cells, blood and lymphatic vessels, lymphoid organs, and metabolites. TME plays a critical role in the recruitment of non-transformed cells, leading to intercellular communication *via* the release of signaling molecules such as cytokines (Figure 3). Wound healing is another critical process in TME that contributes to oncogenic signaling and promotes tumorigenesis (Jin and Jin, 2020). The TME is a complex ecosystem, particularly enriched with various immune components, making it a critical target for cancer therapy. For example, T-cells have been central to the development of immune checkpoint inhibitors and chimeric antigen receptor T-cell (CAR-T) therapies. Additionally, the TME hosts multiple innate immune cells, including myeloid-derived suppressor cells (MDSCs) and natural killer (NK) cells, which are modulated by cytokine signaling and can either support immune surveillance or contribute to immune dysfunction, ultimately leading to tumor progression. Given this complexity, a thorough understanding of the immune landscape of the TME is essential for the development of effective immunotherapeutic strategies (Hinshaw and Shevde, 2019).

In addition to its cellular and molecular components, the TME plays a critical role in regulating epithelial-mesenchymal transition (EMT). During cancer progression and invasion, tumor cells interact with diverse components of TME that switch their phenotype while losing epithelial features. This process helps in the acquisition of mesenchymal and stem cell-like traits that significantly enhance invasiveness and metastatic potential. Evidence reports that TME assists EMT through various signaling pathways and cellular interactions (Suarez-Carmona et al., 2017). Among the various signaling molecules, transforming growth factor-beta (TGF-β) is a key EMT inducer in mediating this process (Yu et al., 2013; de Visser and Joyce, 2023).

Angiogenesis is another crucial TME-regulated mechanism required for advanced cancer progression and metastasis. Angiogenesis ensures a continuous supply of oxygen and nutrients to rapidly growing tumors, thereby facilitating tumor dissemination (Quail and Joyce, 2013). Immune cells present in TME actively contribute to angiogenesis by releasing pro-angiogenic factors that facilitate tumor vascularization. During intravasation and extravasation, macrophages play a significant role in promoting tumor vascularization and facilitating the penetration and traversal of tumor cells across vascular barriers. In systemic circulation, platelets protect tumor cells from immune recognition and channel them to the extravasation site. Additionally, MDSCs and NK cells accumulated in pre-metastatic and metastatic niches support tumor cell dissemination and colonization through modulation of immune responses (Quail and Joyce, 2013). Thus, the TME significantly influences cancer progression *via* EMT, angiogenesis, and immune modulation. Understanding its complexity is crucial for targeted therapies.

## 4 Chemistry of flavonoids

**FIGURE 4:**
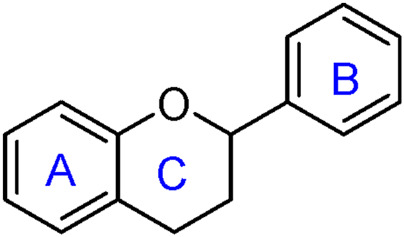
General structure of flavonoid.

**FIGURE 5:**
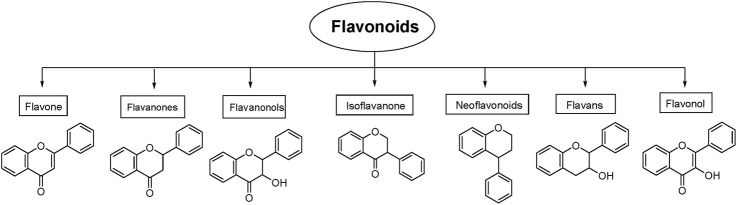
Classification of flavonoids with their basic structure.

Flavonoids are a group of natural compounds with variable phenolic structures. Chemically, flavonoids are based upon a 15-carbon skeleton made up of two benzene rings, referred to as “ring A” and “ring B” respectively (Figure 4) (Sharma et al., 2019). Heterocyclic pyran “ring C” further connects these rings. Interestingly, “isoflavones” are flavonoids with “ring B” linked to “position 3” of “ring C”. The term “neoflavonoids” refers to phytocompounds where “ring B” is connected in the fourth position of “ring C”. Additionally, linking of “ring B” at “position 2” causes the classification of flavonoids into several groups, including flavones (such as luteolin and apigenin), flavonols (such as quercetin, myricetin, and kaempferol), flavanones (such as naringenin and hesperetin), and flavan-3-ols (such as catechin and epicatechin) (Ignat et al., 2011). Generally, the degree of oxidation and the pattern of “ring C” placement vary among distinct types of flavonoids (Figure 5). In contrast, the diversity of specific compounds within a given class of flavonoids is primarily determined by the substitution patterns on rings A and B (Panche et al., 2016). Thus, flavonoids share a common phenolic skeleton but differ structurally, influencing classification and biological activity. Structural diversity underpins their varied anticancer roles.

## 5 Flavonoids and their anticancer potential

Numerous chemotherapeutic agents are available in the market for the treatment of cancers, and several are in the development phase. However, the side effects associated with the non-selectivity towards tumor cells of clinically-used chemotherapeutic agenst have limited their widespread use. These limitations have encouraged research on natural products, which are considered much safer than synthetic agents and have traditionally been used to combat cancers for decades (Demain and Vaishnav, 2011; Khan et al., 2021). Several epidemiological studies documented the reduced risk of cancer by consuming a diet rich in fruits and vegetables (Key, 2011; Wu et al., 2019), which is primarily considered due to the presence of flavonoids. The anticancer potential of flavonoids is attributed to the modulation of enzymes and receptors involved in the signal transduction pathways related to cellular differentiation, proliferation, apoptosis, angiogenesis, metastasis, and reversal of multidrug resistance (Ravishankar et al., 2013). The beneficial effects of flavonoids in cancers are attributed to their antioxidant, immunomodulatory, and anti-inflammatory properties. Natural flavonoids shows anticancer effects by modulating signaling pathways, reducing oxidative stress, and reversing drug resistance. Their safety profile makes them attractive alternatives to synthetic agents.

### 5.1 Influence on tumor microenvironment

Flavonoids have been traditionally employed to carry out potent anticancer activity, incredibly successfully altering the TME. Compounds like epigallocatechin-3-gallate (EGCG) were used to impede TME activity by decreasing the infiltration of tumor-associated macrophages (TAM) in a mouse model of breast cancer (Kopustinskiene et al., 2020). Another flavonoid, anthocyanin, a typical pigment, was noted to increase the levels of anti-inflammatory cytokines such as interleukin-10 (IL-10), enhancing NK cell activation and eliminating tumor cells. Anthocyanins also suppress macrophage infiltration, which affects TME (Lin et al., 2017). Flavonoids possess potent anticancer and antioxidant activities, particularly through their ability to influence tumor regression. One such compound, apigenin, has been shown to profoundly impact the vascular aspects of the TME. Specifically, in a 2014 preclinical study by Mirzoeva et al., treatment with apigenin at 20 µM led to a significant decrease (∼45%, p < 0.01) in vascular endothelial growth factor (VEGF) expression in TGF-β-stimulated human breast cancer cells (MDA-MB-231). This anti-angiogenic effect was mechanistically linked to apigenin’s inhibition of Smad2/3 phosphorylation, as well as reduced activation of Src and downstream FAK/Akt signaling pathways. *In vivo*, mice bearing MDA-MB-231 xenografts treated with 50 mg/kg apigenin exhibited a ∼35% reduction in microvessel density, measured *via* CD31 immunostaining, corroborating the *in vitro* pathway modulation (Mirzoeva et al., 2014; Ou et al., 2024). Simultaneously, naringenin, a related flavonoid, demonstrated complementary TME-modulating effects by promoting anticancer immunity. Kaufman et al. observed that naringenin increased the number of CD169^+^ tumor-associated macrophages, which in turn led to the activation of cytotoxic T cells. It also inhibited lung cancer and melanoma metastasis through suppression of TGF-β/Smad-mediated MMP-2 expression (Kaufman et al., 1997; Liskova et al., 2020; Kawaguchi et al., 2022). These mechanistic and preclinical findings support the proposition that flavonoids such as apigenin and naringenin can effectively modulate TME vascularity and suppress metastatic pathways, reinforcing their promise as bioactive agents against cancer (Dobrzynska et al., 2020). Thus, the results support the translational promise for flavonoids like apigenin and naringenin.

### 5.2 Oxidative stress-mediated pathways

The ROS produced by many cellular organelles such as mitochondria, peroxisomes, and endoplasmic reticulum during cellular metabolism is one of the critical signaling molecules that play prominent roles in various diseases, including cancer (Aggarwal et al., 2019). The body’s defense mechanisms regulate the levels of ROS through the production of specific enzymes like superoxide dismutase, catalase, and glutathione peroxidase, and their nonenzymatic counterparts such as glutathione, ascorbic acid, and α-tocopherol. However, the imbalance between ROS production and antioxidant defense mechanisms results in oxidative stress. An abnormally high level of ROS activates several signaling pathways, ultimately leading to cell death. They react with various biomolecules, *viz.* proteins, lipids, and DNA, to initiate chain reactions, leading to the oxidation of lipids and proteins, DNA damage, and mitochondrial dysfunction (Kaushal et al., 2019).

Flavonoids have dual actions in ROS homeostasis, acting as antioxidants and pro-oxidants (Kopustinskiene et al., 2020). Due to their reducing nature, flavonoids exert an antioxidant effect and exhibit anticancer properties. The underlying mechanisms of the anti-oxidative potential of flavonoids are direct scavenging of ROS, inhibiting oxidases that are responsible for the production of superoxide anion, chelating trace metals, and activating antioxidant enzymes (Procházková et al., 2011; Slika et al., 2022). Interestingly, a study by Lotito and Frei concluded that after consumption of the flavonoid-rich foods, the increase in the plasma antioxidant capacity is not attributed to the flavonoids themselves, but to the consequent increase in uric acid levels (Lotito and Frei, 2006). Filipe and the team showed that the flavonoids inhibited the urate (uric acid) degradation, and it is positively correlated with the inhibition of lipid peroxidation. In a dose-dependent manner, urate inhibited copper-induced lipid peroxidation (Filipe et al., 2001). However, this is contradictory to the studies where flavonoids lead to a decrease in uric acid levels in the patients/subjects/treatment groups with conditions of hyperuricemia, *via* inhibition of xanthine oxidase (Lin S. et al., 2015). In a cross-sectional study by Li and the team, they demonstrated that a diet that is rich in anthocyanins and flavanones significantly lowers uric acid levels and also has a lower incidence of hyperuricemia (Li H. et al., 2023). Acute intake of flavonoid-rich foods like apples leads to an increase in uric acid levels, possibly because of fructose content. Chronic flavonoid intake, especially pure compounds like quercetin, leads to improved renal excretion of uric acid and also a decrease in uric acid synthesis *via* xanthine oxidase inhibition. Thus, flavonoids exhibited a dual role on the uric acid level, which is a context-dependent relationship and needs larger population-based studies for a clear understanding.

Importantly, elevated ROS levels are implicated in cancer drug resistance through various mechanisms such as activating pro-survival pathways (e.g., PI3K/Akt, MAPK, and NF-κB), enhancing DNA repair processes, and inducing the expression of efflux transporters (e.g., P-gp), thus limiting chemotherapy effectiveness. Flavonoids specifically counteract the ROS-mediated drug resistance by directly scavenging ROS, thereby attenuating oxidative stress-induced signaling pathways that promote chemoresistance. Additionally, flavonoids modulate key enzymes such as nicotinamide adenine dinucleotide phosphate (NADPH) oxidases, xanthine oxidase, and cyclooxygenase-2, which are major sources of ROS involved in chemotherapy resistance (Passaniti et al., 2022; Li Y. et al., 2023; Attique et al., 2025). By inhibiting these ROS-producing enzymes, flavonoids effectively attenuate oxidative stress, thereby sensitizing cancer cells to conventional chemotherapeutics (Al-Khayri et al., 2022; Billowria et al., 2024). Recently, kaempferol has been reported to induce ROS-dependent apoptosis in pancreatic cancer cells *via* transglutaminase (TGM2)-mediated Akt/mTOR signaling. Hence, TGM2 is regarded as a promising prognostic biomarker for pancreatic cancer (Wang F. et al., 2021). In addition to oxidative stress-induced apoptosis, kaempferol enhanced the cytotoxic potential of doxorubicin by amplifying ROS toxicity and decreasing the efflux of doxorubicin (Sharma et al., 2007). At the molecular levels, kaempferol suppressed cancer cell proliferation by inhibiting the function of phosphorylated Akt, CyclinD1, CDK4, Bid, Mcl-1, and Bcl-xL, and promoting p-BRCA1, p-ATM, p53, p21, p38, Bax, and Bid expression that trigger apoptosis and S phase arrest (Wu et al., 2018). Similarly, quercetin is also reported to induce apoptosis and ROS-mediated cell death, exert cell cycle arrest at the S and G2/M phases, and display notable anti-metastatic properties (Tubtimsri et al., 2025). In a preclinical study, both quercetin and kaempferol significantly reduced tumor size, demonstrating anticancer activity consistent with their *in vitro* findings (Kusaczuk et al., 2024).

#### 5.2.1 Scavenging of ROS

**FIGURE 6:**
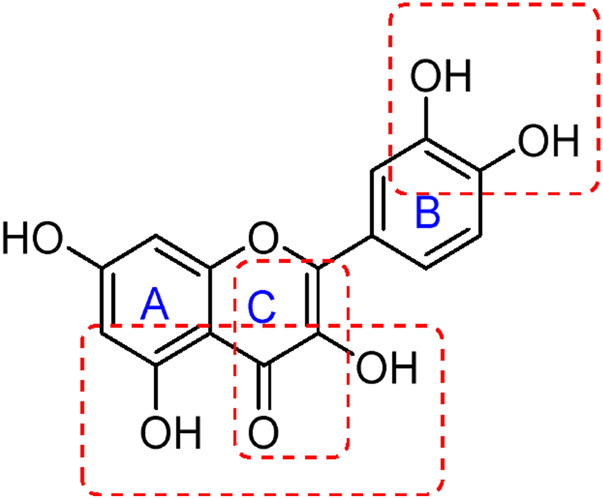
Structural features of flavonoids responsible for direct ROS scavenging abilities.

**FIGURE 7:**
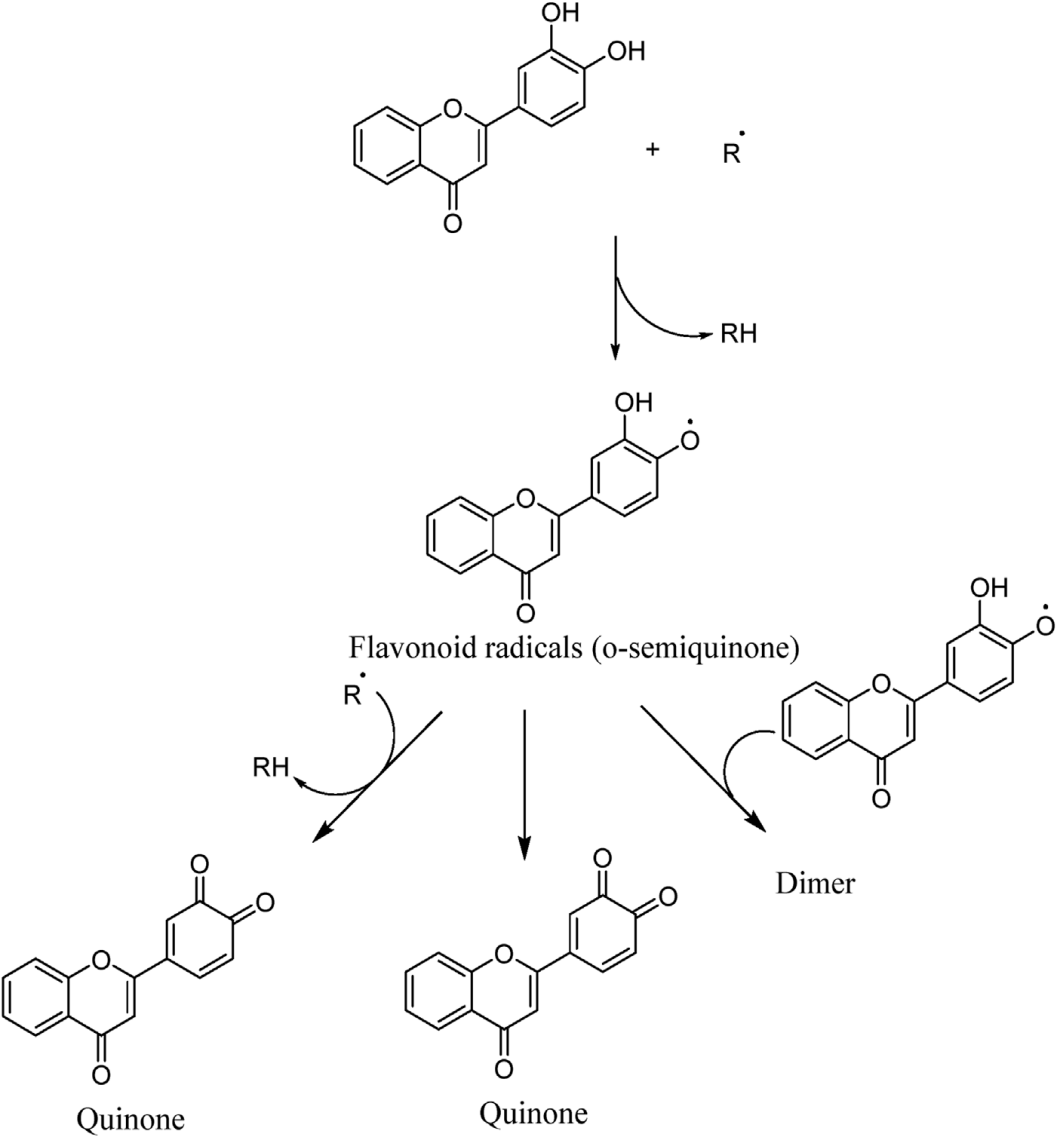
ROS scavenging mechanism of flavonoids (Slika et al., 2022).

The unique structural features of flavonoids, such as hydroxy groups in ring B, 2,3-double bond in conjugation with a 4-oxo functionality in the C ring, and hydroxyl groups at positions 3 and 5 for hydrogen bonding to the oxo group, have empowered the flavonoids with ROS scavenging abilities (Figure 6). Flavonoids scavenge the highly oxidizing free radicals by directly donating hydrogen atoms and forming flavonoid radicals (o-semiquinone) (Rolt and Cox, 2020). These radicals, in turn, depending upon their structures, further undergo either coupling with another oxidizing radical or donate another hydrogen atom to form quinone or form a dimeric product by dimerization with another flavonoid radical (Figure 7) (Slika et al., 2022).

#### 5.2.2 Inhibition of ROS-producing enzymes

In addition to directly scavenging ROS species, inhibition of ROS-producing enzymes such as xanthine oxidase, cyclooxygenase, and NADPH oxidase is a significant attribute of the antioxidant ability of flavonoids. Xanthine oxidase is a critical enzyme that catalyzes the oxidative hydroxylation of hypoxanthine and xanthine to uric acid, which subsequently reacts with molecular oxygen, leading to the generation of ROS, primarily superoxide anion radical or hydrogen peroxide (Lin S. et al., 2015). Moreover, inhibition of xanthine oxidase increases the levels of hypoxanthine and xanthine, which in turn inhibit *de novo* purine biosynthesis *via* a feedback mechanism, an essential treatment pathway for some of the antineoplastic drugs (Rahaman et al., 2022). Several flavonoids such as quercetin, chrysin, luteolin, kaempferol, myricetin, and isorhamnetin are potent inhibitors of xanthine oxidase (Chang et al., 1993; Nagao et al., 2014), and therefore decrease the risk of oxidative stress-induced diseases. Among the various flavonoids, planar flavones and flavonols with a 7-hydroxyl group have been reported to possess potent xanthine oxidase inhibitor activity as compared to nonplanar flavonoids like isoflavones and anthocyanidins (Nagao et al., 2014). Hydroxyl groups at positions 5 and 7 in ring A are essential for binding to amino acid residues of the active site of the enzyme xanthine oxidase *via* hydrogen bonding. In addition, a double bond at the 2,3-position in ring C and the presence of keto functionality at the 4-position are required for inhibitory activity. Furthermore, pi-pi stacking between ring B and the amino acid residue of xanthine oxidase enhances the active site binding (Van Hoorn et al., 2002; Mathew et al., 2015). Blumeatin is a predominantly non-coplanar flavonoid, featuring an over-twisted torsion angle of approximately −94.6° between rings A and B. Contrary to expectations, this structural deviation correlates with enhanced radical scavenging activity, likely by influencing the accessibility and reactivity of its hydroxyl groups, although it may still reduce efficacy in some enzyme-binding contexts (Altunayar-Unsalan and Unsalan, 2024; Li et al., 2024).

Overexpression of cyclooxygenase (COX)-2 has been implicated in the development and progression of various human cancers and is associated with the resistance of cancer cells to conventional chemotherapy and radiotherapy (Shim et al., 2003; Pang et al., 2016). Expression of COX-2 is modulated by various vital regulators such as peroxisome proliferator-activated receptor gamma (PPAR-γ) (KnopfovÁ and ŠMarda, 2010), NK-κB (Lim et al., 2001), and TGF-β (Saha et al., 1999). The interplay between flavonoids and these key regulators suppresses the COX expression, showing beneficial effects in many diseases (Ramachandran et al., 2012; Li et al., 2020; Yan et al., 2020). Mutoh et al. reported quercetin as the most potent suppressor of COX-2 transcription, while catechin and epicatechin showed weak activity (Mutoh et al., 2000). The presence of the 4-oxo group in ring C, 3,4-dihyroxyl groups in ring B, and low electron density of the oxygen atom in the 7-hydroxyl group in ring A are considered to be important structural features in flavonoids for the suppression of COX-2 transcriptional activity (Mutoh et al., 2000; Hou et al., 2005). In several studies, flavonoids have been reported to show chemopreventive action *via* suppression of the COX enzyme (Sakata et al., 2003; Ye et al., 2004; Al-Fayez et al., 2006; Chen et al., 2008). In contrast to xanthine oxidase and other enzymes that generate ROS as byproducts, NADPH oxidases are the only enzymes dedicated to ROS generation as their primary product (Maraldi, 2013). Flavonoids have been identified as more potent NADPH oxidase inhibitors than ROS scavengers (Jimenez et al., 2015; Luo et al., 2019). Flavonoids having hydroxyl groups in ring B and saturated 2,3 bond in C ring exhibited NADPH oxidase inhibitor activity, which is potentiated by the presence of vicinal hydroxy-methoxy arrangement at an aromatic ring (Steffen et al., 2008).

#### 5.2.3 Chelating trace metals

Metal ions such as Fe and Cu are reported to catalyze the production of ROS species, which have detrimental effects on many diseases. In the presence of hydrogen peroxide, iron, even in small traces, catalyzes hydroxyl radical formation *via* the Fenton reaction (Lyngsie et al., 2018). Copper reacts with hydrogen peroxide *via* a free radical mechanism to form superoxide and hydroxyl radicals (Angelé-Martínez et al., 2017). Oxidative stress-induced cellular redox imbalance has been implicated in various cancer cells. By their metal-chelating properties, flavonoids play a significant role in metal overload diseases. Quercetin, rutin, and catechin have more substantial complexation properties toward iron and zinc ions than copper ions (Chamani et al., 2016). In another study, flavones (apigenin, luteolin, kaempferol, quercetin, myricetin, and rutin), isoflavones (daidzein and genistein), flavanones (taxifolin, naringenin, and naringin), and a flavanol (catechin) exhibited higher reducing capacity for copper ions than for iron ions (Mira et al., 2009). Morin, combined with another flavonoid (naringin), exhibits synergistic activity in the chelation of lead ions (Adhikari et al., 2018).

Moreover, metal ion-flavonoid complexes, besides retaining the antioxidant activities, are much more effective free radical scavengers than the free flavonoids, which is assumed to be due to the acquisition of additional superoxide dismutating centers (de Souza and De Giovani, 2013). Structural features of flavonoids revealed that unsaturation at the 2,3-position, a hydroxyl group at the three-position, and a catechol group in ring B showed better iron-reducing activity (Mira et al., 2009). In flavonols and flavones, the most efficient copper chelation sites were the 3-hydroxy-4-keto group (ring C) and 5,6,7-trihydroxyl group (ring A), respectively, as the 3′,4′-dihydroxyl group (ring B) was associated only with weak activity (Ríha et al., 2014). Chelation of metal ions by flavonoids renders them inactive in generating radicals. Also, the generated radicals are intercepted by flavonoids themselves, making them an essential compound in managing metal-overload-induced oxidative stress-based diseases.

#### 5.2.4 Activation of antioxidant enzymes

Another possible mechanism by which flavonoids act is by activating antioxidant enzymes, suppressing pro-oxidant enzymes, and stimulating the production of antioxidant enzymes and phase II detoxification enzymes (Kopustinskiene et al., 2020). On exposure to ROS, various genes encoding detoxifying and antioxidative stress enzymes/proteins are coordinately induced, which is regulated by antioxidant-responsive element (ARE) or electrophile-responsive element (EpRE). The coordination of ARE/EpRE encodes various phase II detoxifying enzymes such as NAD(P)H-quinone oxidoreductase 1, glutathione-*S*-transferase, and UDP-glucuronosyl transferase (Itoh et al., 2004). Nuclear factor erythroid-derived 2-related factor 2 (Nrf2), sequestered in the cytoplasm by Kelch-like ECH-associated protein 1 (Keap1), regulates the transcription of ARE-driven genes. On exposure of cells to ARE inducers, Nrf2, after dissociation from Keap1, is translocated to the nucleus. Here, it heterodimerizes with a small Maf protein and binds to ARE, eventually leading to transcriptional regulation of target genes (Lee and Surh, 2005; Uruno and Motohashi, 2011). Flavonoids promote the translocation of Nrf2 to the nucleus, activating detoxifying enzymes that facilitate the detoxification of carcinogens (Moon et al., 2006). Genistein, a dietary flavonoid, has been reported to show antioxidant defense by induction of Nrf2 and phase II detoxification gene expression *via* extracellular signal-related protein kinases and its pathway (Zhai et al., 2013). Another class of flavonoids, anthocyanins, has been demonstrated to show antioxidant capacity in cell line studies by activating ARE upstream of genes involved in antioxidation and detoxification (Shih et al., 2007).

In summary, flavonoids regulate ROS homeostasis by scavenging radicals, inhibiting ROS-producing enzymes, chelating metals, and activating antioxidant defenses. This dual role contributes to cancer prevention and sensitization to therapy.

### 5.3 Anti-inflammatory and immunomodulatory-mediated pathways

Chronic inflammation plays a significant role in cancer initiation, promotion, and progression (Coussens and Werb, 2002). Inflammatory mediators like cytokines, ROS, and reactive nitrogen species derived from tumor-infiltrating immune cells induce epigenetic alterations in premalignant lesions and silence tumor suppressor genes in the initial phase of tumor development (Multhoff et al., 2012). Various oncogenic transcription factors, such as NK-κB and STAT3, are often activated by inflammatory mediators (primarily due to the elevated production of IKK-activating cytokines, including tumor necrosis factor (TNF) and IL-1). In contrast, oncogenes such as Ras and Myc can initiate inflammatory responses (Taniguchi and Karin, 2018). Tumor-associated inflammation transforms tumor-specific immune cells from anti-tumorigenic to pro-tumorigenic, suppressing the anti-tumor immune response (Grivennikov and Karin, 2010). During the promotion phase, various chemokines and cytokines secreted by immune cells act as survival and proliferation factors for malignant cells. During the tumor progression and metastasis, tumor and immune cells produce further cytokines and chemokines, increasing cell survival, motility, and invasiveness (Grivennikov and Karin, 2010; Multhoff et al., 2012). Alterations in many of the cell signaling pathways, particularly those in levels of kinases such as MAPK and protein kinases that regulate cell proliferation and differentiation, have been implicated in the inflammatory processes. Silencing or abnormal activation of these kinases or their downstream transcription factors can result in uncontrolled cell growth, leading to malignant transformation (Fresco et al., 2006; García-Lafuente et al., 2009).

**FIGURE 8:**
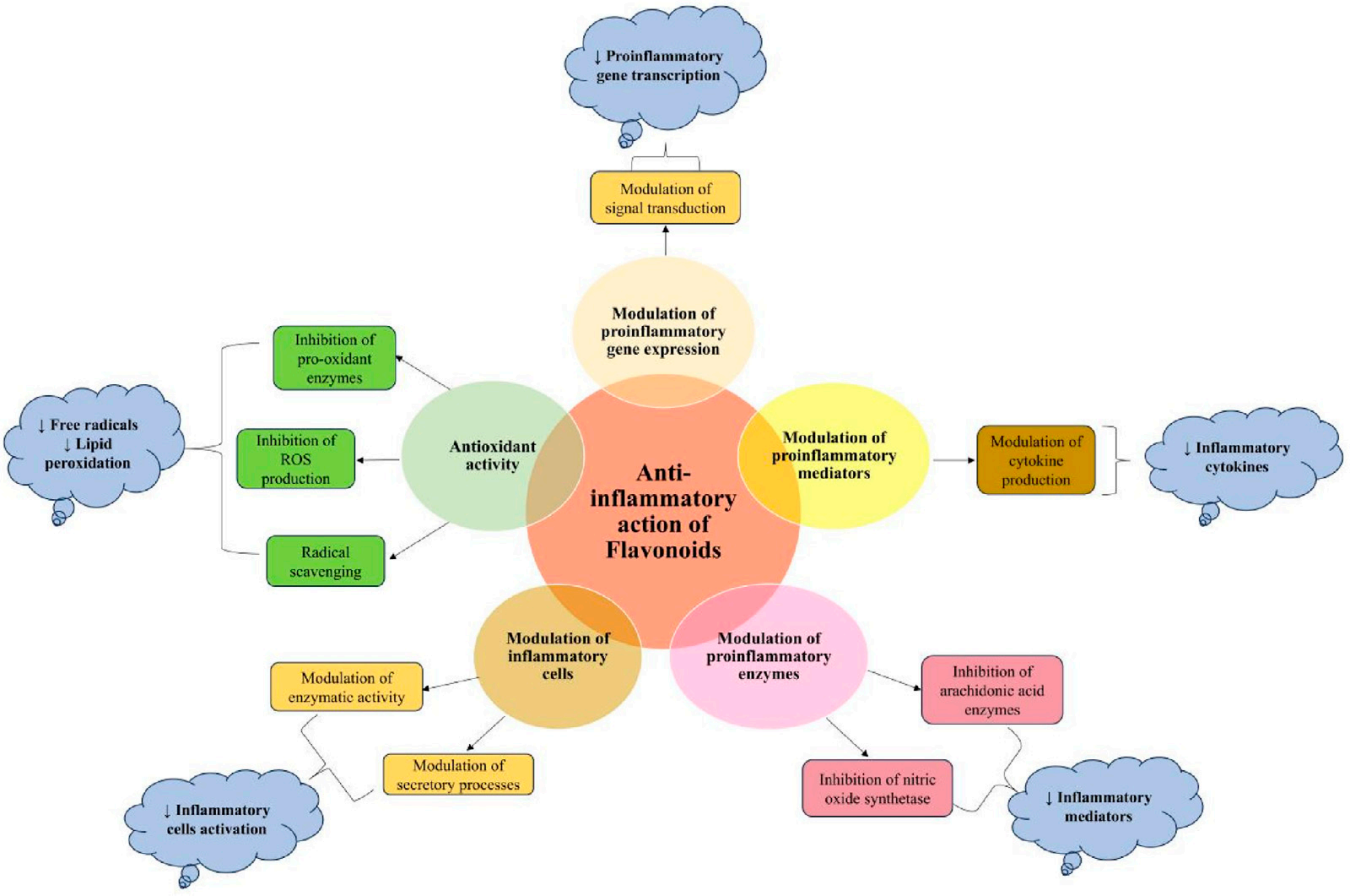
Mechanistic pathways of anti-inflammatory action of flavonoids (García-Lafuente et al., 2009).

Increasing scientific documentation regarding the anti-inflammatory potential of flavonoids (Wei et al., 2005; Feng et al., 2012) has led to their exploration of several diseases, including cancer. Mechanistically, the anti-inflammatory potential of flavonoids is attributed to their antioxidant and radical scavenging activities, regulation of immune cells, modulation of enzymes involved in the biosynthesis of inflammatory mediators, and regulation of proinflammatory gene expression (García-Lafuente et al., 2009) (Figure 8). Several *in vitro* and preclinical studies have demonstrated the potential role of flavonoids in preventing carcinogenesis. Some of these studies and their mechanism have been summarized in Table 1. Concludingly, flavonoids modulate immune and inflammatory pathways, suppressing tumor-promoting signals while enhancing chemoprevention. Their mechanisms span enzyme regulation, cytokine modulation, and signaling interference.

**TABLE 1:** *In vitro* and preclinical studies demonstrating anti-inflammatory mechanisms implicated in chemoprevention.

Mechanism	Flavonoid	Chemical structure	Class	Cancer model	Study type	References
COX-II inhibition	Apigenin	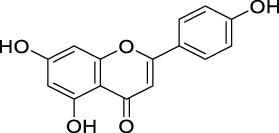	Flavones	Skin cancer	*In vitro*	Van Dross et al. (2007)
	Genistein	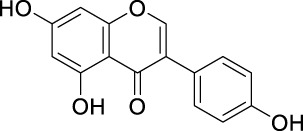	Isoflavones	Breast cancer	*In vitro*	Horia and Watkins (2006)
	Naringin	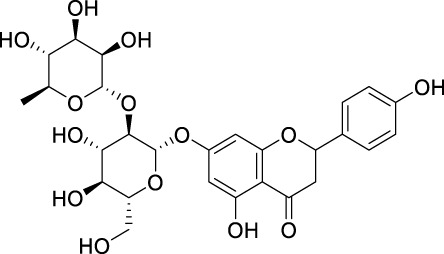	Flavanone	Colon cancer	*In vivo*	Vanamala et al. (2006)
	Tricin	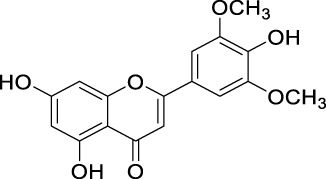	Flavones	Intestinal adenoma	*In vivo*	Cai et al. (2005)
PK inhibition	Apigenin	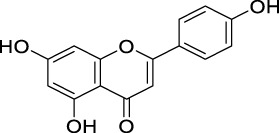	Flavones	Skin cancer	*In vitro*	Huang et al. (1996)
	Luteolin	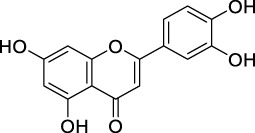	Flavones	Pancreatic tumor	*In vitro*	Lee et al. (2002)
				Lung cancer, breast cancer, liver cancer, and pancreatic cancer	*In vitro*	Huang et al. (1999)
	Quercetin	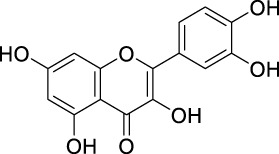	Flavonols	Pancreatic tumor	*In vitro*	Lee et al. (2002)
				Lung cancer, breast cancer, liver cancer, and pancreatic cancer	*In vitro*	Huang et al. (1999)
MAPK inhibition	Apigenin	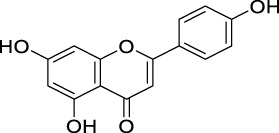	Flavones	Prostate cancer	*In vitro*	Shukla and Gupta (2014)
				Breast cancer	*In vitro*	Yin et al. (2001)
				Bladder cancer	*In vitro*	Xia et al. (2018)
NF-κB suppression	Apigenin	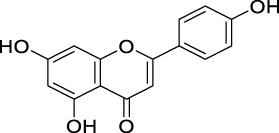	Flavones	Prostate cancer	*In vitro*	Shukla and Gupta (2004)
	Genistein	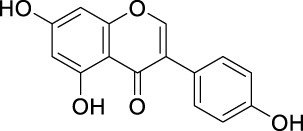	Isoflavones	Prostate cancer	*In vitro*	Davis et al. (1999), Li and Sarkar (2002)
	Morin	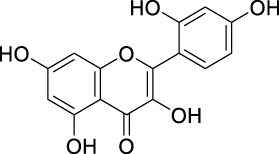	Flavonols	Bladder cancer	*In vitro*	Shin et al. (2017)
				Lung cancer	*In vitro*	Manna et al. (2007)
Modulating ROS production and interfering with MAPK and NF-κB signaling pathways	Quercetin	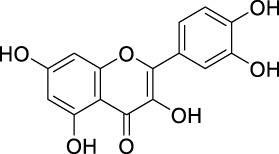	Flavonols	Prostate cancer	*In vitro*	Ward et al. (2018)
Antioxidant activity	Quercetin	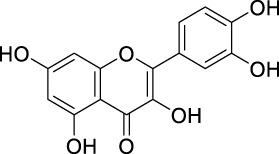	Flavonols	Liver cancer	*In vivo*	Seufi et al. (2009)
				Lung cancer	*In vivo*	Kamaraj et al. (2007), Alzohairy et al. (2021)
	Hesperidin	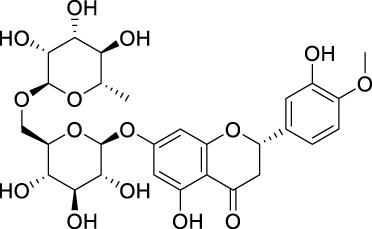	Flavanone	Lung cancer	*In vivo*	Kamaraj et al. (2008)
	Chrysin	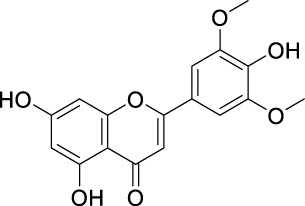	Flavones	Lung cancer	*In vivo*	Kasala et al. (2016b)
	Luteolin	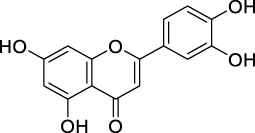	Flavones	Lung cancer	*In vivo*	Kasala et al. (2016a)

Abbreviations: COX, cyclooxygenase; MAPK, Mitogen-activated protein kinase; NF-κB, Nuclear factor-κB; PK, protein kinase; ROS, reactive oxygen species.

## 6 Emerging flavonoid subclasses with translational promise

While quercetin, apigenin, and kaempferol dominate historical discussion, several less-covered subclasses now show compelling anticancer signals together with formulation paths that address exposure limits. Below we highlight four such leads and outline mechanisms, ADME opportunities, and near-term study designs.

### 6.1 Isorhamnetin (flavonol; 3′-*O*-methylquercetin)

A 2025 state-of-the-art review comprehensively mentioned isorhamnetin’s multi-hallmark actions, including cell cycle arrest (G1/S, G2/M), apoptosis (mitochondrial/caspase), anti-angiogenesis, anti-metastasis, and immune/TME modulation, and collates nanoformulation-based approaches that improve delivery and bioavailability (Rana et al., 2025). These data support rational combinations (for example, with cytotoxic or targeted agents) and motivate PK-anchored designs that track target-pathway readouts (e.g., PI3K/AKT/mTOR, NF-κB) (Rana et al., 2025).

### 6.2 Prunin (naringenin-7-*O*-glucoside; flavone glycoside)

The first focused review in 2025 positions prunin as emerging anticancer candidate that modulates cell cycle and apoptosis pathways, with early evidence for combination use and nanoformulations to overcome solubility/bioavailability limits (Rana and Mumtaz, 2025). Given its glycosidic nature and gut microbiota conversion to prunetin, studies should integrate metabolite profiling with exposure-response endpoints (Rana and Mumtaz, 2025).

### 6.3 Taxifolin/dihydroquercetin (flavanonol)

A recent comprehensive review details direct antitumor activity (proliferation/migration inhibition, apoptosis, and anti-angiogenesis), chemosensitization (e.g., P-gp downregulation), immunomodulation, and a strong cytoprotective profile against several chemotherapy-based toxicities, while explicitly noting PK limitations and delivery/chemistry solutions (Sarg et al., 2024). These features make taxifolin a pragmatic adjunct in combination regimens, provided exposure is optimized (Sarg et al., 2024).

### 6.4 Dihydromyricetin/ampelopsin (dihydroflavonol)

A recent mechanistic review summarizes broad anticancer actions, including anti-proliferative, pro-apoptotic, anti-invasion, autophagy modulation, and redox regulation, across diverse array of cancers. This review further highlights instability/low bioavailability as translation bottlenecks that are being addressed with nano- and crystal-based systems (Xia and Zhu, 2024). Early combination data with standard chemotherapies further justify exposure-controlled and biomarker-rich trials (Xia and Zhu, 2024).

These subclasses broaden the field beyond “the usual suspects”, and critically arrive with formulation blueprints (such as nanoparticles, nanogels, and lipid carriers), that raise tumor deposition and reduce systemic peaks.

## 7 Clinical trials evaluating the anticancer potential of flavonoids

Numerous epidemiologic studies have demonstrated that certain flavonoids, as well as diets or foods rich in flavonoids, are beneficial in preventing cancer development and its progression. Moreover, flavonoids reduce the established high-risk indicators associated with cancer (Baby et al., 2021). However, additional clinical trial studies are required to assess the efficacy of flavonoids in people with a range of characteristics, including age, gender, disease severity and stage, prior treatment history, and comorbid medical conditions (Kikuchi et al., 2019).

Flavonoids, like those found in tea and coffee, are consumed worldwide, and several research studies have demonstrated their positive benefits on human wellbeing, including their ability to prevent cancer. According to an analysis by Yang and Hong of case-controlled and prospective cohort studies that were published in 2008, the consumption of green tea reduced the cancer risk in 39 cases of colon, breast, esophageal, kidney, lung, ovarian, pancreatic, stomach, and prostate cancers, whereas 46 cases indicated no risk reduction. These observations imply that green tea may help to avoid certain cancers (Yang and Hong, 2013; Suzuki et al., 2016; Hayakawa et al., 2020). Therapeutic potential of flavonoids significantly differs with various factors like age, gender, and disease state. Geriatric patients exhibit enhanced benefits due to increased oxidative stress patterns but decreased bioavailability from various metabolic changes with age-related factors. The flavonoid’s phyloestrogenic activity is associated with various gender differences. In the cases of various disease states like diabetes/inflammation may enhance the antioxidant effects of flavonoids by making a mediation in gut microbiota-mediated metabolism. Such factors as age, gender, and diseases need to be considered for flavonoid-based therapeutic approaches (Del Rio et al., 2013; Fraga et al., 2019; Favari et al., 2024; Hu et al., 2025).

To assess the effectiveness of flavonoids in cancer therapy, Bisol et al. looked at some clinical trial studies (Phases II and III). The U.S. Food and Drug Administration (FDA) states that phase II clinical studies are carried out to evaluate a medicine’s effectiveness in treating an illness, and phase III studies are carried out to confirm that a possible drug is superior to the gold standard treatment option. In all, 1 phase III and 22 phase II clinical trials that employed flavonoid compounds as a single entity or in combination with other therapies to treat lymphoid cancer that were published by January 2019 were chosen for study. It was shown that flavopiridol is the most frequently administered flavonoid compound (at a dose of 50 mg/m^2^ IV) for most of the cases. Flavonoids demonstrated sophisticated positive consequences for lymphoid and hematopoietic tissues than for solid tumors (4 patients with complete response (CR) and 21 with partial response (PR) among 525 patients in 12 trials), despite the relatively low rate of CR or partial response PR with any administration protocol (Bisol et al., 2020). Some of the flavonoids whose anticancer potential in human subjects has been evaluated or are currently under investigation have been summarized in Table 2. 60 trial entries spanning ∼17 flavonoid categories/combos are covered where the most studied agents are catechin, genistein, quercetin, and EGCG. While there was strong emphasis on prostate malignancies followed by multiple studies related to breast, lung, bladder, colon/colorectal, and skin cancer, there were trials related to ovarian, cervical, esophageal, kidney, head and neck premalignancy, glioma/glioblastoma, multiple myeloma, and follicular lymphoma. When considering the phase trials status, predominantly phase 2, with some phase 1/early phase 1, and only a handful of phase 3 studies. Further, studies include purified flavonoids, botanical preparations, and drug-flavonoid combinations, reflecting an adjunct/chemo-sensitization emphasis. While epidemiological studies and early-phase trials suggest promise, clinical outcomes remain variable and context-dependent. More large-scale, controlled studies are needed.

**TABLE 2:** Clinical studies on the anticancer potential of flavonoids [https://www.clinicaltrials.gov/(Accessed August, 2025)].

Flavonoid	Cancer	Phase	Subject characteristics	Status	NCT
Anthocyanins	Breast cancer	Not applicable	Age: ≥18 years Sex: Female	Completed	NCT02195960
Anthocyanins	Colorectal adenoma	Not applicable	Age: 18–75 years Sex: All	Unknown	NCT01948661
Catechin	Skin cancer	Phase 2/Phase 3	Age: ≥18 years Sex: All	Completed	NCT02029352
Catechin	Bladder cancer	Phase 2	Age: ≥18 years Sex: All	Completed	NCT00666562
Catechin	Breast cancer	Phase 2	Age: 50–70 years Sex: Female	Completed	NCT00917735
Catechin	Breast cancer	Phase 1	Age: 21–65 years Sex: Female	Completed	NCT00516243
Catechin	Breast cancer	Not applicable	Age: ≥18 years Sex: Female	Completed	NCT00949923
Catechin	Cervical cancer	Phase 2	Age: ≥18 years Sex: Female	Completed	NCT00303823
Catechin	Esophageal cancer	Phase 1	Age: ≥18 years Sex: All	Completed	NCT00233935
Catechin	Lung cancer	Phase 2	Age: 45–74 years Sex: All	Completed	NCT00573885
Catechin	Lung cancer	-	Age: 18–70 years Sex: All	Available	NCT01317953
Catechin	Ovarian cancer	Phase 2	Age: All age groups Sex: Female	Completed	NCT00721890
Catechin	Prostatic intraepithelial neoplasia	Phase 2	Age: 30–80 years Sex: Male	Completed	NCT00596011
Catechin	Prostate cancer	Phase 1	Age: ≥18 years Sex: Male	Completed	NCT00459407
Catechin	Prostate cancer	Not applicable	Age: 21–120 years Sex: Male	Completed	NCT00253643
Catechin	Skin cancer	Not applicable	Age:18–65 years Sex: All	Completed	NCT01032031
Catechin	Liver cancer with cirrhosis	Phase 1	Age: ≥18 years Sex: All	Active, not recruiting	NCT03278925
Catechin	Prostate cancer	Phase 2	Age: ≥18 years Sex: Male	Recruiting	NCT04300855
Epigallocatechin gallate	Prostate cancer	Phase 2 Phase 3	Age: 50–69 years Sex: Male	Completed	NCT01105338
Epigallocatechin gallate	Colorectal cancer	Phase 2	Age: 50–80 years Sex: All	Completed	NCT01360320
Epigallocatechin gallate	Colon cancer	Not applicable	Age:19–85 years Sex: All	Completed	NCT02321969
Epigallocatechin-3-gallate	Lung cancer	Phase 2	Age: ≥18 years Sex: All	Unknown	NCT02577393
Epigallocatechin-3-gallate	Breast cancer	Phase 2	Age: 18–70 years Sex: All	Unknown	NCT02580279
Epigallocatechin gallate	Colon cancer	Early Phase 1	Age: ≥18 years Sex: All	Recruiting	NCT02891538
Epigallocatechin gallate	Urothelial Carcinoma	-	Age: 20–80 years Sex: All	Unknown status	NCT01993966
Genistein	Prostate cancer	Phase 2/Phase 3	Age: All age groups Sex: Male	Completed	NCT00584532
Genistein (Isoflavone G-2535)	Bladder cancer	Phase 2	Age: ≥18 years Sex: All	Completed	NCT00118040
Genistein	Breast cancer	Phase 2	Age: ≥25 years Sex: Female	Completed	NCT00290758
Genistein	Breast cancer	Phase 2	Age: 18–120 years Sex: Female	Completed	NCT00244933
Genistein	Pancreatic cancer	Phase 2	Age: 21–120 years Sex: All	Completed	NCT00376948
Genistein	Cancer	Phase 1/Phase 2	Age: 2–20 years Sex: All	Completed	NCT02499861
Genistein	Colorectal cancer	Phase 1/Phase 2	Age: ≥18 years Sex: All	Completed	NCT01985763
Genistein	Lung cancer	Phase 1/Phase 2	Age: ≥18 years Sex: All	Completed	NCT01628471
Genistein	Breast cancer Endometrial cancer	Phase 1	Age: 45–70 years Sex: Female	Completed	NCT00099008
Genistein	Cancer	Phase 1	Age: All age groups Sex: All	Completed	NCT00001696
Genistein	Kidney cancer	Early phase 1	Age: ≥18 years Sex: All	Completed	NCT00276835
Genistein	Bladder cancer	Phase 2	Age: ≥18 years Sex: All	Active, not recruiting	NCT01489813
Genistein	Prostate cancer	Phase 2	Age: ≥18 years Sex: Male	Unknown status	NCT00546039
Genistein	Pancreatic cancer	Phase 1/Phase 2	Age: ≥18 years Sex: All	Unknown	NCT01182246
Genistein	Leukemia, Lymphoma	Phase 1	Age: up to 80 years Sex: All	Unknown	NCT00004858
Isoquercetin	Kidney cancer	Phase 1/Phase 2	Age: ≥18 years Sex: All	Unknown	NCT02446795
Luteolin	Tongue cancer	Early Phase 1	Age: All Sex: All	Unknown	NCT03288298
Polyphenols	Breast cancer	Not Applicable	Age: ≥18 years Sex: All	Completed	NCT03482401
Polyphenon E	Lung cancer	Phase 2	Age: 40–80 years Sex: All	Completed	NCT00363805
Polyphenon E	Premalignant Lesions of the Head and Neck	Phase 1	Age: ≥18 years Sex: All	Completed	NCT01116336
Quercetin	Prostate cancer	Phase 2	Age: ≥18 years Sex: Male	Completed	NCT03493997
Quercetin	Prostate cancer	Phase 1	Age: 40–75 years Sex: Male	Completed	NCT01912820
Quercetin	Prostate cancer	Phase 2	Age: ≥18 years Sex: Male	Recruiting	NCT04252625
Quercetin	Squamous cell carcinoma	Phase 2	Age: ≥2 years Sex: All	Recruiting	NCT03476330
Quercetin	Follicular lymphoma	Phase 2	Age: ≥18 years Sex: All	Unknown	NCT00455416
Quercetin	Oral cancer	Phase 2	Age: All Sex: All	Unknown	NCT05456022
Quercetin	Breast cancer	Early Phase 1	Age: 18–70 years Sex: Female	Not yet recruiting	NCT05680662
Quercetin	Pancreatic ductal adenocarcinoma	Not Applicable	Age: ≥18 years Sex: All	Unknown	NCT01879878
Quercetin and Genistein	Prostate cancer	Not Applicable	Age: 18–65 years Sex: Male	Unknown	NCT01538316
Hesperidin and Diosmin	Breast Cancer	Phase 3	Age: ≥18 years Sex: Female	Not yet recruiting	NCT06811220
Genistein	Pediatric Cancer	Phase 2	Age: 1 Year to 21 Years (Child, Adult) Sex: All	Terminated	NCT02624388
Rutin combined with Tislelizumab and GC (Gemcitabine and Cisplatin)	Platinum-refractory Muscle-invasive Bladder Cancer	Phase 1	Age: 18 Years and older (Adult, Older Adult) Sex: All	Recruiting	NCT06916494
Silibinin	Glioblastoma	Not applicable	Age: 18 Years–99 Years (Adult, Older Adult) Sex: All	Not yet recruiting	NCT06964815
Green Tea + Quercetin + Docetaxel	Castration-resistant Prostate Cancer	Phase 1/Phase 2	Age: 18 Years and older (Adult, Older Adult) Sex: Male	Recruiting	NCT06615752
Dasatinib, Quercetin, Fisetin and/or Temozolomide	Glioma	Early phase 1	Age: 18 Years and older (Adult, Older Adult) Sex: All	Not yet recruiting	NCT07025226
Quercetin + Dasatinib + CAR-T Therapy	Relapsed or Refractory Multiple Myeloma	Phase 2	Age: 18 Years and older (Adult, Older Adult) Sex: All	Recruiting	NCT06940297

## 8 ADME properties of anticancer flavonoids

Here we summarize what currently limits oral/systemic exposure for flavonoids and which delivery/chemistry knobs reliably move the PK needle. Once flavonoids are taken orally, it becomes crucial to comprehend their pharmacodynamics. Additionally, it is beneficial to analyse the available structure-activity relationship (SAR) data to identify the pharmacological activities associated with flavonoids. In conjunction with SAR attributes, the pharmacokinetics, biotransformation, and metabolism of flavonoids play a significant role in determining their pharmacological effects. Further investigation is required to elucidate factors such as absorption rate, pharmacokinetics, metabolite characterization, and the physiological effects of these metabolites to establish a correlation between the SAR of flavonoids and their impact on human nutrition and medicine. Flavonoids are commonly found in food items in the form of *O*-glycosidic compounds, which are conjugated with glucose, glucorhamnose, arabinose, galactose, or rhamnose units (Hammerstone et al., 2000). When checked on PubMed using search terms including flavonoid, ADME, and cancer, it yielded 67 articles. We read the abstracts of each of these articles and then selected studies relevant to this theme.

The β-linkages in these sugars make them resistant to hydrolysis by pancreatic enzymes. However, the process of β-hydrolysis is carried out by specific intestinal microbiota. Such microbiota include *Peptococcus*, *Peptostreptococcus*, and *Clostridia*, which ferment these sugars in the colon (Cummings and Macfarlane, 1991). Specific enzymes like lactase, phlorizin hydrolase, classified as β-endoglycosidase, are known to deglycosylate flavonoids, creating sites for conjugation (Leese and Semenza, 1973; Day et al., 2000). Flavonoids such as quercetin-3-glucoside, luteolin-7-glucoside, and kaempferol-3-glucoside are absorbed and hydrolyzed in the small intestine through the action of β-glucosidases (Gopalan et al., 1992).

Flavonoids, a diverse group of compounds known for their abundance and potency, have been extensively studied to gain insights into their absorption and metabolism (Hollman et al., 2009). The absorption kinetics of flavonoids can vary significantly due to different sugars and functional groups in the flavan nucleus. Several factors, including dosage, administration route, vehicle of administration, colon microbiota, diet, food matrix, and sex differences, contribute to the variability in flavonoid absorption (Erlund, 2001).

In addition to the hydrolysis of flavonoid glycosides, the cecal microflora also plays a role in breaking down monomeric flavonoids into monophenolic acids. For instance, intestinal bacteria can cleave the C3-C4 bond of the heterocyclic structure, resulting in the production of quercetin metabolites such as 3,4-dihydroxy-phenylacetic acid and phloroglucinol (Winter et al., 1991).

A previous study revealed that rats exposed to rutin produced specific compounds, including 3,4-dihydroxytoluene, phenylacetic acids, and 3-(m-hydroxyphenyl) propionic acid, in their urine (Baba et al., 1983). The hydrolysis of rutinosides (quercetin-3-rutinoside) by colonic microflora was found to be more significant compared to quercetin glycoside (quercetin-3-glucoside), explaining the lower bioavailability of rutin observed in human studies (Olthof et al., 2000). In human subjects, the absorption of quercetin in the small intestine was found to increase to 52% when a glucose unit was present in the molecular structure, compared to 24% for the aglycone unit and 17% for rutin-based compounds. This increase in absorption suggests that quercetin glucoside facilitates interaction with epithelial glucose transporters, leading to a more rapid uptake and improved bioavailability of glucosides following ingestion (Gee et al., 1998).

The biodegradation of larger flavone molecules into more minor, low molecular weight compounds is necessary to cross the intestinal epithelium. According to Déprez et al., dimers and trimers of procyanidin can move across the epithelium of the small intestine. Since these molecules typically consist of (+) catechin and (−) epicatechin subunits, catechins are likely the primary by-products of degradation (Déprez et al., 1999). The bacterial colony facilitates this degradation process in the cecum, and the lower pH in the stomach (Spencer et al., 2000; Groenewoud and Hundt, 2009). The hydrolysis of proanthocyanidin oligomers into catechin dimers and free catechins takes approximately 3.5 h in the gastric environment. After three catechin units, the degradation rate increases proportionally to the degree of polymerization. Although it has been suggested that catechins are responsible for the pharmacological effects of proanthocyanidins with high molecular weight, the duration of 3.5 h or more exceeds the average human gastric emptying rate of 30–90 min. Therefore, the influence of acid hydrolysis is undoubtedly less significant than subsequent metabolic processes (Winter et al., 1991).

In a study carried out by Doostdar et al.*,* it was discovered that the flavones acacetin and diosmetin possess the ability to inhibit the ethoxy resorufin *O*-dealkylase (EROD) activity of two specific cytochrome P450 enzymes, namely CYP1B1 and CYP1A. The selectivity of these enzymes was primarily influenced by substitutions at the 3′ and 4′ positions of the flavonoid structures involving hydroxy and/or methoxy functional groups. Interestingly, other flavonoids such as naringenin, eriodictyol, and homoeriodictyol exhibited poor inhibitory effects on human CYP1A EROD activity (Doostdar et al., 2000).

Hesperetin and homoeriodictyol were found to perform *O*-demethylation of hesperetin to produce eriodictyol in both human CYP1A1 and CYP1B1. Furthermore, homoeriodictyol demonstrated the ability to inhibit human CYP1B1 selectively. It was observed that hesperetin could not be metabolized by human cytochromes CYP1A2 or CYP3A4. *In vitro* studies using human liver and intestinal microsomes indicated that luteolin primarily undergoes glucuronidation at the seventh position in liver cells. In contrast, in intestinal cells, it occurs at the third and fourth positions. The conjugation of luteolin with intestinal microsomes was approximately three times more prevalent than with liver microsomes. Individual testing of the enzymes revealed variations in the efficiency of glucuronidated luteolin, with certain forms displaying higher efficiency than others (Hostetler et al., 2017).

A recent study by Semwal et al. revealed that specific structural features of myricetin, such as the C2 and C3 double bond, the aromatic ring B at position C2, and the hydroxy groups in ring B, may contribute to its cytotoxic properties (Semwal et al., 2016). Previously, it was believed that quercetin was excreted in feces due to its inability to be absorbed in the intestine. However, another research by Murota et al. demonstrated that quercetin is absorbed in the intestine and converted into metabolites (Murota et al., 2002). The transportation of these quercetin metabolites involves the lymphatic system, as observed in a study (Terao et al., 2008).

Furthermore, a study by Moon et al. indicated that females’ regular consumption of onions leads to the accumulation of quercetin metabolites in the bloodstream and various tissues. Specifically, after 1 week of treatment, the total plasma concentration of these metabolites reached 0.6 μM. Rutin exhibits limited bioavailability, which hinders its biological effectiveness. Several drug delivery systems have been developed to overcome this challenge and enhance rutin’s bioavailability (Moon et al., 2000). These systems include nanoparticulate formulations, sulphonation and carboxylation of rutin, enzymatic oligomerization, and cyclodextrin complexation, which improve its aqueous solubility.

Additionally, phospholipid complexation, enzymatic and chemical acylation, and nanoparticulation techniques are employed to enhance the lipid solubility of rutin (Choi et al., 2021). Flavonoids, such as myricetin, kaempferol, quercetin, apigenin, and luteolin, are commonly found in plants and are widely consumed. On average, individuals consume approximately 23 mg/day of these antioxidant flavonoids, surpassing the intake of well-known antioxidants like β-carotene (2–3 mg/day) and vitamin E (7–10 mg/day). Additionally, this intake represents around one-third of the average intake of vitamin C (70–100 mg/day) (Hertog et al., 1993).

Among the various flavonoids, quercetin is the most significant contributor to the dietary consumption of flavonoids. It is predominantly obtained from apples and onions (Knekt et al., 2000; Gibellini et al., 2011). Both preclinical and clinical studies have indicated that quercetin glucosides, cinnamate conjugates, and flavonols are readily absorbed in the small intestine (Olthof et al., 2003; Cermak et al., 2007). However, quercetin galactosides, quercetin, rutin, and naringenin are not absorbed in the intestine. While the exact mechanism of absorption remains unclear, the transport of flavonoids across membranes plays a crucial role in their bioavailability in plants and animals. Further research suggests that ATP-dependent pumps and ATP-independent transporters contribute to this process (Passamonti et al., 2009).

Genistein, an isoflavone, is commonly found in high-soy diets. It has gained significant recognition due to its potential in preventing and treating different cancer types. Many research studies have demonstrated that genistein exhibits anticancer properties, specifically against ovarian cancer. However, its bioavailability, which refers to the amount of the compound that reaches the bloodstream and target tissues, is limited (Lee et al., 2012). Yang et al. conducted mechanistic studies to investigate the factors influencing the ADME of genistein. In terms of absorption, they found that genistein is efficiently absorbed in the small intestine but undergoes significant metabolism in the liver, resulting in lower levels reaching the systemic circulation. The absorption process is influenced by various factors, including the presence of food, the gut microbiota, and the transporters responsible for moving genistein across cell membranes. Regarding distribution, genistein has been detected in various tissues, indicating its ability to reach different target sites in the body. However, its distribution may be affected by factors like protein binding and tissue-specific uptake mechanisms (Yang et al., 2012).

Metabolism studies revealed that genistein undergoes extensive biotransformation in the liver, primarily through phase II enzymes. These enzymes attach other molecules to genistein, making it more water-soluble and facilitating its excretion from the body. Additionally, the gut microbiota also plays a role in the metabolism of genistein. It is primarily excreted through the bile into the feces, with a small portion eliminated in the urine. The researchers highlight the importance of understanding the factors affecting genistein’s ADME to optimize its therapeutic potential and develop effective strategies for its delivery. Understanding these mechanisms is crucial for harnessing the potential health benefits of genistein and developing strategies to enhance its therapeutic efficacy (Yang et al., 2012).

In laboratory studies, flavonoids have shown significant anticancer effects in refractory non-small cell lung cancer (NSCLC) models. However, their effectiveness in clinical applications depends on ADME factors within the body (Manach and Donovan, 2004). Consequently, researchers have recognized the importance of pharmacokinetics as a critical factor in the anticancer properties induced by flavonoids. Investigations have been conducted to assess the ingestion and excretion of flavonoids to understand this aspect (Gugler et al., 1975). The findings from these pharmacokinetic studies, summarized in Table 3, offer valuable insights for guiding effective flavonoid treatments in various types and stages of cancers. Flavonoid absorption, metabolism, and bioavailability are influenced by structural features, gut microbiota, and formulation strategies. Optimizing ADME is essential for clinical translation.

**TABLE 3:** Summary of research on the pharmacokinetics of flavonoids *via* oral administration.

Flavonoids	Dosage	Model	T_max_ (h)	T_1/2ke_ (h)	C_max_ (μM)	AUC_0−t_ (μM*h)	Bioavailability (%)	References
Apigenin	13.51 mg·kg^−1^	Sprague–Dawley rats	0.50 ± 0.01	2.11 ± 0.03	42 ± 2	659 ± 25	NA	Wang et al. (2021b)
Naringenin	150 mg per person	Human	3.17 ± 0.74	3.0	15.76 ± 7.88	67.61 ± 24.36	NA	Clementi et al. (2021)
	600 mg per person		2.41 ± 0.74	2.65	48.45 ± 7.88	199.05 ± 24.36		
Genistein	100 mg·kg^−1^ BIO 300 (nanosuspension of genistein)	*Macaca mulatta*, (Nonhuman primate, *n* = 4, named as NHP1–4)	4	2.37	10,400	60,788	NA	Cheema et al. (2021)
			2	2.88	6,330	38,775		
			2	4.09	5,150	54,492		
			2	2.19	5,560	29,703		
Luteolin	100 mg·kg^−1^	Sprague–Dawley rats	4.83 ± 1.56	2.2 ± 0.2	3,070 ± 720	10,183 ± 1,483	26 ± 6	Lin et al. (2015a)
Isoliquiritigenin	20 mg·kg^−1^	SD rats and Kunming mice	0.5	4.4 ± 0.42	1,100 ± 80	4,500 ± 210	29.9	Qiao et al. (2013)
	50 mg·kg^−1^		0.5	4.4 ± 0.62	4,400 ± 410	8,600 ± 500	22.7	
	100 mg·kg^−1^		0.5	4.8 ± 0.18	14,300 ± 1,730	25,400 ± 630	33.6	
Silibinin	140 mg SMEDDS capsule per person	Human	0.80 ± 0.44	1.91 ± 1.85	812.43 ± 434.07	658.80 ± 266.23	NA	Sornsuvit et al. (2018)
Flavokawain B	10 mg·kg^−1^	Sprague–Dawley rats	1.00 ± 0.55	2.76 ± 1.19	265.2 ± 117.6	1,009.8 ± 601.3	NA	Yang et al. (2019)
Hesperetin	135 mg solid dispersion capsule	Human	4.00	3.12	623.37	3,411.50	3.26	Kanaze et al. (2007)
Narirutin	96.4 mg	Human	5.0 ± 0.45	NA	0.20 ± 0.04	351 ± 90	NA	Manach and Donovan (2004)
Hesperidin	50 mg/kg	Rat— Sprague Dawley	8.0 ± 2.8	NA	1.92 ± 1.03	NA	NA	Jin et al. (2010)
Quercetin (from quercetin glycoside-rich onion powder)	∼100 mg quercetin aglycone equivalents, single oral dose (100 g apple sause +47.5 g onion powder)	Human (healthy adults)	2.0 ± 1.7	14.8 ± 4.8	0.904 (273.2 ± 93.7 ng/mL)	2,340 ± 713 ng·h/mL	NA	Lee and Mitchell (2012)
Quercetin (from quercetin glycoside-rich apple powder)	∼100 mg quercetin aglycone equivalents, single oral dose (100 g apple sause +85 g apple peel)	Human (healthy adults)	2.9 ± 2.0	65.4 ± 80.0	63.8 ± 22.4 ng/mL	843 ± 371 ng·h/mL	NA	Lee and Mitchell (2012)
Quercetin (from quercetin glycoside-rich mixed powder)	∼100 mg quercetin aglycone equivalents, single oral dose (100 g apple sause +42.5 g apple peel +23.7 g onion powder)	Human (healthy adults)	2.4 ± 1.5	18.7 ± 6.8	136.5 ± 45.8 ng/mL	1,415 ± 580 ng·h/mL	NA	Lee and Mitchell (2012)
Baicalein (tablets)	Day 1: 600 mg single oral dose	Human (healthy adults)	2.7 ± 1.06	14.91 ± 9.91	845.20 ± 1,122.98 ng/mL	4,380.65 ± 3,225.68 h ng/mL	NA	Li et al. (2021a)
	Day 10: 600 mg (In between day 4–9: 3 times daily)	Human (healthy adults)	1.71 ± 1.04	11.29 ± 4.2	1,322.50 ± 901.79 ng/mL	12,384.96 ± 9,226.29 h ng/mL	NA	Li et al. (2021a)

Abbreviations: T_max_, Time to peak drug concentration; T1/2ke, Elimination plasma half-life; C_max_, Concentration Maxima; AUC0−t, Area under the concentration-time curve from dosing; NSCLC, non-small cell lung cancer.

## 9 Undesired toxicity of anticancer flavonoids

We also outline actionable safety practices-DDI screens (especially CYP3A4, exposure-tuning *via* delivery, and dose/schedule choices to preserve efficacy while minimizing risk.

### 9.1 Quercetin

One of the most essential dietary phytoconstituents among bioflavonoids is quercetin. Its name comes from the Latin word “quercetum”, which means “oak forest”. Its formal name, 2-(3, 4-dihydroxyphenyl)-3,5,7-trihydroxy-4H-chromen-4-one, is known chemically (Miltonprabu, 2019; Magar and Sohng, 2020). It has a bitter flavor and is common in meals, drinks, and dietary supplements. When the enzymes phenylalanine ammonia-lyase, cinnamate-4-hydroxylase, and 4-coumaroyl-CoA ligase are present, phenylalanine is converted to 4-coumaroyl-CoA *via* the phenylpropanoid route to produce quercetin (Lesjak et al., 2018).

The enzymatic action of 7,2′-dihydroxy-4′-methoxyisoflavanol synthase further combines 4-coumaroyl-CoA to malonyl-CoA (3 molecules), which results in the production of tetrahydroxychalcone. Additionally, the enzyme chalcone isomerase changes tetrahydroxychalcone into naringenin, which is then changed back into eriodictyol by the enzyme flavonoid 3′-hydroxylase (F3′H). Furthermore, the enzymatic action of flavanol synthase transforms eriodictyol into dihydroquercetin, followed by creating quercetin (Lesjak et al., 2018). Due to its antioxidant and therapeutic properties, which include anticancer, anti-inflammatory, and anti-obesity actions, quercetin has attracted much interest from scientists recently (Devappa et al., 2015; Ezzati et al., 2020; Khazdair et al., 2021). Additionally, it has demonstrated its effectiveness against various conditions, including gout, pancreatic cancer, asthma, and disorders similar to Alzheimer’s disease (Ulusoy and Sanlier, 2019). Interestingly, quercetin has been linked to lower cataract risk due to dermatological problems and diabetes complications (Mishra and Rizvi, 2012).

**FIGURE 9:**
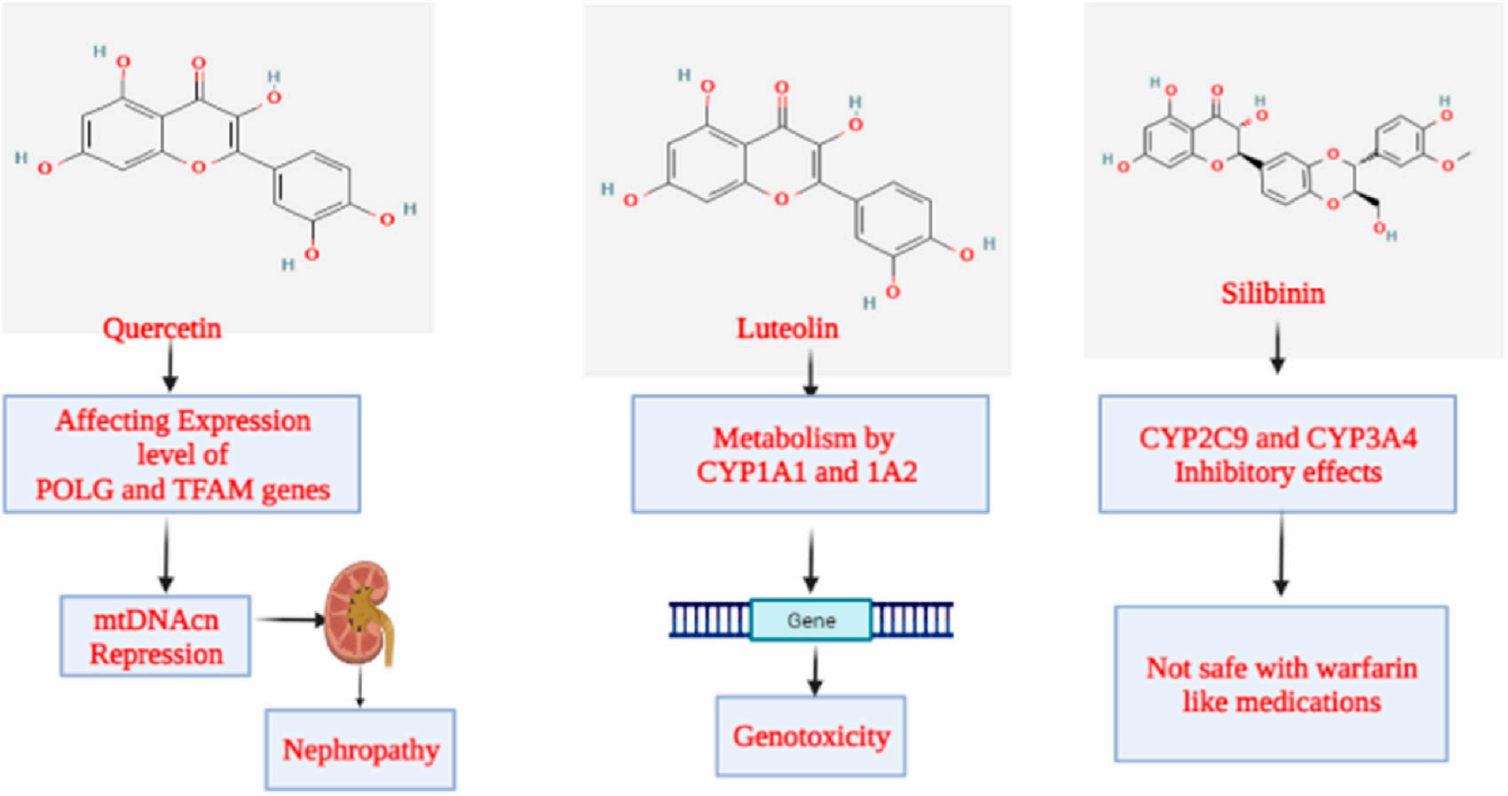
Mechanism of toxicity exhibited by flavonoids.

Regarding the toxicity parameters, specific points need to be discussed. Chen et al. surprisingly discovered using an ICR murine model that animals subjected to 7 Gy whole-body irradiation (TBI) demonstrated general *in vivo* toxicity following the administration of quercetin (100 mg/kg PO). However, this result was not seen in mice that received TBI alone. They employed a real-time qPCR to analyze the mitochondrial DNA copy number (mtDNAcn) by amplifying the MTRNR1 (12S rRNA) gene in the murine bone marrow to comprehend the role of changes in mitochondrial biogenesis. Reverse transcription-quantitative PCR was also used to quantify the levels of mRNA produced in the tissue from the polymerase gamma (POLG), polymerase gamma 2 (POLG2), and mammalian mitochondrial transcription factor A (TFAM) genes. Brought on by the suppression of POLG expression and the overexpression of TFAM, the findings indicate that the overall toxicity was partially linked to the reduction in mtDNAcn; POLG2 expression that was left intact did not appear to contribute to toxicity (Chen et al., 2014). According to the US National Toxicology Program, the high quercetin dosage from the 6-month intervention has already contributed to developing nephropathy. Based on the research mentioned above, the issue of whether large doses of quercetin ingestion in humans and animals might exacerbate the underlying kidney-damaging processes is raised (Figure 9). Although no significant detrimental effects of quercetin on kidney function have been shown in human intervention trials, quercetin use by individuals with renal failure should be interpreted cautiously (Andres et al., 2018).

### 9.2 Luteolin

The class “flavones” of flavonoids includes luteolin, often referred to as 3′,4′,5,7-tetrahydroxyflavone, another naturally occurring phytoconstituent that is extensively distributed. It has three rings with a carbon double bond at position 2-3 and a C6-C3-C6 structure. The 3rd, 5th, and 7th carbon locations of luteolin are known to have hydroxyl groups, respectively (Weng and Yen, 2012). Furthermore, it has been shown that the hydroxyl moieties and the double bond at positions 2nd and 3rd are necessary for luteolin’s bioactivities. Additionally, luteolin has been linked to glycosylation in plants, which leads to additional hydrolysis and the luteolin release (Aziz et al., 2018). Luteolin is a harmless heat-stable phytocompound. Several papers have mentioned its anti-carcinogenic, anti-inflammatory, and antioxidant activities (Choi et al., 2014; Tuorkey, 2016). Additionally, it has demonstrated the ability to prevent diabetes and cardiovascular disorders by triggering the immune system. It functions as a scavenger of nitrogen and oxygen species that may directly or indirectly impair cellular integrity (Bharti et al., 2014). Luteolin has anti-estrogenic properties that inhibit cell growth, making it a powerful antiproliferative agent (Bharti et al., 2014). Li et al. investigated the cytotoxicity and genotoxicity that luteolin causes and the part that several CYPs play in luteolin’s bioactivation. Human lymphoblastoid TK6 cells and TK6-derived cell lines (CYP1A1, 1A2, 1B1, 2A6, 2B6, 2C8, 2C19, 2D6, 2E1, 3A4, 3A5, and 3A7) that each stably express a single human cytochrome P450 were used in that study. Treatments with luteolin for 4–24 h caused concentration-dependent cytotoxicity, apoptosis, DNA damage, and chromosomal damage. Then, we discovered that TK6 cells transduced with CYP1A1 and 1A2 greatly boosted luteolin-induced cytotoxicity and genotoxicity as determined by the high-throughput micronucleus assay. In addition, CYP1A1-and 1A2-expressing cells showed a substantial increase in important DNA damage and apoptosis indicators, such as cleaved PARP-1, cleaved caspase-3, and phosphorylated histone 2AX (H2A.X), when compared to empty vector controls. After 24 h, TK6 cells bio-transformed the bulk of luteolin into diosmetin, a less toxic *O*-methylated flavone; the presence of CYP1A1 and 1A2 partly inhibited this process, according to LC-MS/MS analysis. These findings collectively show that CYP1A1 and 1A2 metabolism increased the toxicity of luteolin *in vitro* (Figure 9). These studies provide additional evidence for the effectiveness of TK6 cell system in identifying the precise CYPs accountable for chemical bio-activation and toxicity potential (Li X. et al., 2021).

### 9.3 Silibinin

Silibinin, a flavonoid derived from the parent plant *Silybum marianum* (L.) Gaertn. (Milk thistle) is one of the significant flavonoids obtained from plants. A group of phytocompounds known as “flavonolignan” is generally present in milk thistle (Li X. et al., 2021). However, silybin, also known as silibinin, the first recognized component in this complex, combines two diastereomers: silybin A and silybin B. A semi-purified, commercially accessible portion of silibinin is Silymarin (Samu et al., 2004; Tahsin et al., 2019). It is interesting to note that the production of silibinin requires the coupling of two crucial substances, namely taxifolin and coniferyl alcohol. Numerous pharmacological effects have been reported, mainly concerning fatty liver conditions such as non-alcoholic fatty liver, steatohepatitis, and alcoholic liver cirrhosis (Shaker et al., 2010; Zhu et al., 2018). According to Soleiman et al., the Asteraceae family includes therapeutic plants like milk thistle [*S. marianum* (L.) Gaertn.]. The main component of milk thistle extract is Silymarin, a combination of flavonolignans such as silybin, which is Silymarin’s most potent component. It is mainly recognized for its hepatoprotective properties. Its safety is crucial since studies have revealed further therapeutic benefits, including those against cancer, Alzheimer’s disease, Parkinson’s disease, and diabetes. Animal tests show no significant toxicity. *Salmonella typhimurium* strains were mutagenic to Silymarin in the presence of metabolic enzymes. At a concentration of 100 μM, silybin, silydianin, and silychristin were not cytotoxic or genotoxic. Silymarin is well tolerated, even at high dosages of 700 mg three times per day for 24 weeks, and is safe in humans at therapeutic levels. There were some gastrointestinal discomforts, such as diarrhea and nausea. According to a clinical investigation, Silymarin is safe during pregnancy, and there were no malformations. As a result, prenatal care should be taken, and additional research, especially in humans, is required. Silymarin does significantly affect cytochromes P450 and has few DDIs. Studies have shown that Silymarin should be used cautiously with medications with a limited therapeutic window (Figure 9) (Soleimani et al., 2019).

### 9.4 Genistein

**FIGURE 10:**
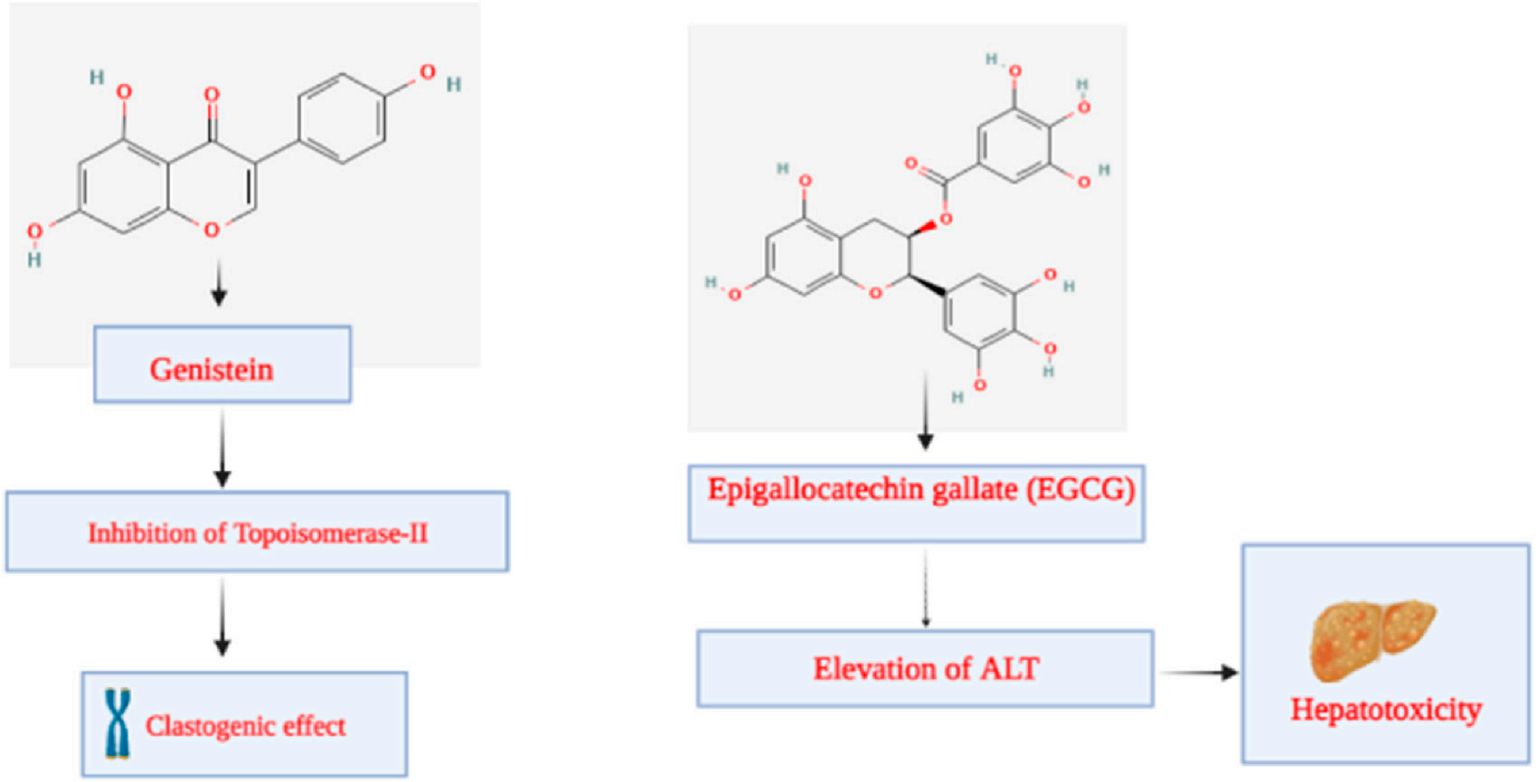
Toxicity elicited by genistein and epigallocatechin gallate.

Genistein is a member of the isoflavone subclass. It is a phytoestrogen from plants in the Fabaceae family, including *Vicia faba* L., *Glycine* max (L.) Merr., *Pueraria lobata* (Wild.), *Lupinus albus* L., and others. According to its chemical name, 5,7-dihydroxy-3-(4-hydroxyphenyl) chromene-4-one, genistein has a structure comparable to mammalian estrogens (Dixon, 2002). It consists of 15 carbons, each of which has two aromatic rings, referred to as “ring A” and “ring B”, which are connected to “ring C”, a carbon pyran ring. In addition to the oxo group at the fourth position of ring C, this phytoconstituent’s carbon skeleton/structure also includes a double bond between the second and third locations. Recent investigations have demonstrated the therapeutic efficacy of genistein in preventing cancer, osteoporosis, cardiovascular disorders, and postmenopausal issues (Marini et al., 2007; Thangavel et al., 2019). As an anti-inflammatory agent, it has also demonstrated a crucial function in inflammatory disorders (Chen et al., 2020). According to recent research, genistein may play a significant role in neurodegenerative diseases through an autophagy-dependent mechanism (Chen et al., 2020). In the studies regarding the genetic toxicity of genistein conducted by McClain et al., it was revealed that genistein was found to be neither mutagenic nor clastogenic *in vivo* in the mouse and rat micronucleus test or in the *S. typhimurium* assay. Genistein increased the number of primarily tiny colonies in the mouse lymphoma experiment, showing clastogenic properties. This finding is consistent with research published on genistein’s inhibitory effect on topoisomerase II, which is known to cause chromosomal damage with a threshold dose response (Figure 10) (Michael McClain et al., 2006).

### 9.5 Epigallocatechin gallate

Epigallocatechin gallate (EGCG), also known as epigallocatechin-3-gallate, is an ester of epigallocatechin and gallic acid and is a member of the catechin family of polyphenols. It has been noted that catechins are responsible for the tea [*Camellia sinensis* (L.) Kuntze]’s ability to prevent cancer (Chu et al., 2017). The primary phytochemical in green tea, EGCG, is one of the several catechins. It is interesting to note that the dried leaves of green tea contain the most EGCG overall, followed by those of white tea and black tea, respectively (Jin et al., 2015).

Researchers have discovered a wide range of health benefits of drinking tea in recent years, many of which are attributable to the presence of EGCG. It has demonstrated favorable activity against various illnesses, including cancer, rheumatoid arthritis, cardiovascular disease, and inflammation (Lecumberri et al., 2013; Chourasia et al., 2021). Lambert et al. studied the hepatotoxicity of EGCG. They looked at how highly-dosed EGCG affected the livers of male CF-1 mice. EGCG elevated plasma alanine aminotransferase (ALT) by 138-fold and decreased survival by 85% after a single dosage (1,500 mg/kg, i.g.). The hepatotoxic reaction was exacerbated by EGCG administered once daily. Following two 750 mg/kg once-daily doses of EGCG, plasma ALT levels rose by 184 times. Following therapy with EGCG, moderate to severe hepatic necrosis was seen. The hepatotoxicity of EGCG was linked to oxidative stress, which included elevated plasma 8-isoprostane (9.5-fold rise), hepatic metallothionein, and gamma-histone 2AX protein expression. Hepatic lipid peroxidation was also enhanced (5-fold). Monocyte chemoattractant protein-1 and plasma IL-6 were likewise elevated by EGCG. According to those studies, mice’s livers are damaged by greater bolus dosages of EGCG. Given the growing popularity of green tea dietary supplements, which may provide significantly greater plasma and tissue concentrations of EGCG than tea drinks, further research is needed to understand the dose-dependent hepatotoxic effects of EGCG and the underlying processes (Figure 10) (Lambert et al., 2010).

In conclusion, high flavonoid intake may lead to the development of various adverse effects, which include primarily hepatotoxicity, disruption of the endocrine system, pro-oxidant activity, and other endocrine disruptions. Exclusively high doses of catechins from tea are associated with liver injury. Isoflavones act mainly as endocrine system disrupters. Flavonoids also produce their pharmacological action by blocking various cytochrome P450 enzymes and increasing the risk of toxicity. Various side effects such as gastrointestinal disturbances and renal damage were also reported in supplements with highly concentrated flavonoids. This implies the need for highly concentrated research focusing on the toxicity profile of flavonoids (Galati and O'Brien, 2004; Deng et al., 2025).

While flavonoids show therapeutic benefits, toxicity profiles vary across compounds and conditions, necessitating careful dose optimization and safety evaluation.

## 10 Future perspectives

Studying the anticancer flavonoids’ PK, drug-likeness, and toxicological properties offers considerable potential for developing new treatments. Because of their antioxidant, anti-inflammatory, and cell-signaling-modulating actions, flavonoids, a varied collection of naturally occurring substances found in plants, have been researched for their potential anticancer qualities. The following are some potential outcomes of studying these issues in the future.

### 10.1 Enhanced drug design and development

Researchers may find it helpful to better grasp the PK parameters of anticancer flavonoids to develop more accurate and successful pharmacological formulations. With this knowledge, flavonoid derivatives may be developed with a higher bioavailability and tissue-specific dispersion, boosting their therapeutic potential (Kopustinskiene et al., 2020).

### 10.2 Optimized dosing strategies

Pharmacokinetic investigations can guide the development of ideal dose regimens for anticancer flavonoids. This is essential for the body to remain at therapeutic levels while reducing possible toxicity and negative consequences (Liskova et al., 2021).

### 10.3 Prediction of drug-likeness

Evaluation of flavonoids’ chemical and physical characteristics is necessary to identify whether they can develop into new drugs. To prioritize flavonoids with a better chance of being effective in developing pharmaceuticals, mathematical and machine learning methods can be employed to forecast how well these molecules conform to the features of currently used medications (Dias et al., 2021).

### 10.4 Toxicological insights

It is crucial to investigate and ensure about the toxicological characteristics of anticancer flavonoids during clinical trials and ultimate clinical application. Establishing acceptable dosage ranges and identifying probable side effects/off-targets can be aided by thorough toxicological analyses (Choudhari et al., 2019).

### 10.5 Combination therapy

By understanding these compounds’ ADMET characteristics, researchers can investigate the potential of anticancer flavonoids in combination therapies with other cancer treatments, such as chemotherapy or targeted therapies. This might improve therapy effectiveness and possibly lower the dosages needed for conventional anticancer medications, reducing their adverse effects, and for reversing the anticancer drug resistance (Choudhari et al., 2019).

### 10.6 Personalized medicine

Researchers can work toward creating individualized/personalized therapy methods by examining the variation in PK and toxicological reactions to anticancer flavonoids among people. This could entail adjusting doses following a patient’s genetic composition and other aspects, resulting in more efficient and secure therapies (Hu et al., 2017).

### 10.7 Regulatory approval

Comprehensive PK and toxicological data are required to assess the safety and efficacy of novel anticancer treatments for compliance with regulatory organizations like the FDA. Clinical translation can be facilitated by thorough research in several fields, speeding up the approval process. Future research of flavonoids will focus on pharmacogenomics and the gut microbiome will personalize the treatment because the ethnographic variation in metabolism and biotransformation in the abdomen will influence the toxicity and efficacy. Moreover, we can suggest that combination therapies with existing chemo-medications will reduce the various adverse reactions and as well as drug resistance *via* different synergistic pharmacological mechanisms. In addition to that, advancing various PK understanding, proper toxicological evaluations will transform the flavonoids for developing novel nutraceutical-based approaches for anti-cancer drug therapy (de Sousa Silva et al., 2022; Farhan et al., 2023; Tomar et al., 2024).

Strategies such as improved drug design, optimized dosing, combination therapies, and personalized medicine approaches are critical for advancing flavonoid-based cancer treatment.

## 11 Conclusion

The study results on the pharmacokinetics, drug-likeness, and toxicological characteristics of anticancer flavonoids provide insight into their potential as attractive candidates for cancer treatment. Comprehensive studies have revealed that flavonoids, a group of polyphenolic chemicals abundantly found in various plant sources, have complex PK profiles that affect how quickly they are absorbed, distributed, metabolized, and excreted by the body. The benefits of flavonoids have been emphasized in several studies, including their capacity to regulate various drug-metabolizing enzymes, transporters, and signaling pathways. Because of their adaptability, they have the potential to be used as adjuvants to conventional chemotherapy, enhancing therapeutic efficacy and minimizing side effects through synergistic interactions.

Additionally, the drug-likeness evaluation highlights the structural variety of flavonoids, which have benefits and drawbacks. Although the intricacy of these compounds and their polyphenolic makeup can prevent them from being optimized as conventional small-molecule medicines, novel drug delivery methods and packaging techniques have been developed to overcome these drawbacks. These strategies seek to increase these drugs’ bioavailability, stability, and targeted delivery to tumor locations, increasing their clinical viability (Rosales and Fabi, 2023).

Extensive studies into the anticancer flavonoids’ safety profiles have shown their typically low toxicity and appropriate safety margins regarding toxicological characteristics. However, it is crucial to recognize that their toxicity profiles may be affected by the dose-response relationship, possible drug interactions, and individual variances in metabolism. Determine their long-term safety and potential side effects through rigorous preclinical and clinical testing (Zhang et al., 2020).

The potential flavonoids that have cytotoxic activity, which mainly consist of quercetin, kaempferol, genistein, and epigallocatechin gallate. These compounds possess a wide range of pharmacokinetic advantages when these molecules will be formulated as nanoformulations and also prodrugs that can increase bioavailability. These molecules possess a very favorable ADMET profile and will also be considered for further exploration in anticancer research. On considering the toxicological parameters, these compounds possess less systemic toxicity. Major developments in various drug delivery systems, the *in silico* ADMET profiling *via* various computational approaches can improve the safety and efficacy of anticancer flavonoids. On considering all the above facts, flavonoid molecules have a better clinical translational potential as effective anticancer drugs.

The core of the investigation into anticancer flavonoids is the confluence of PK insights, drug-likeness considerations, and toxicological evaluations. Despite the progress that has been achieved, there is still a need for pharmacologists, chemists, toxicologists, and clinicians to work together transdisciplinarily to bridge the gap between laboratory findings and clinical applications. Further studies should concentrate on improving the PK profiles of flavonoid compounds, enhancing their molecular mechanisms of action, and carrying out extensive clinical trials to determine their genuine therapeutic potential in cancer treatment.

In conclusion, the extensive investigation of the PK, drug-likeness, and toxicological characteristics of anticancer flavonoids reveals a terrain rife with prospects and difficulties. This study deepens our understanding of these substances and opens the door to novel therapeutic approaches that use their unique qualities to advance cancer treatment.

## Glossary

ADMET: Absorption, distribution, metabolism, excretion, and toxicity
ADME: Absorption, distribution, metabolism, and excretion
ARE: Antioxidant-responsive element
AUC0−t: Area under the concentration-time curve from dosing
ALT: Alanine aminotransferase
CYPs: Cytochromes P450s
COX: Cyclooxygenase
CR: Complete response
**C**_**max**_: Concentration Maxima
EMT: Epithelial-mesenchymal transition
EpRE: Electrophile-responsive element
EGCG: Epigallocatechin gallate
FDA: Food and Drug Administration
F3′H: Flavonoid 3′-hydroxylase
Keap1: Kelch-like ECH-associated protein 1
**LD**_**50**_: Lethal dose that kills 50% of the test organisms
MAPK: Mitogen-activated protein kinase
MDR: Multidrug resistance
MDSCs: Myeloid-derived suppressor cells
mtDNAcn: Mitochondrial DNA copy number
TFAM: Mitochondrial transcription factor A
NADPH: Nicotinamide adenine dinucleotide phosphate
NF-κB: Nuclear factor-κB
NSCLC: Non-small cell lung cancer
PK: Protein kinases
PR: Partial response
POLG: Polymerase gamma
POLG2: Polymerase gamma 2
ROS: Reactive oxygen species
SAR: Structure-activity relationship
STAT3: Signal transducer and activator of transcription-3
TAM: Tumor-associated macrophages
TME: Tumor microenvironment
TNF: Tumor necrosis factor
**T**_**max**_: Time to peak drug concentration
T1/2ke: Elimination plasma half-life
